# Mutation burden and other molecular markers of prognosis in colorectal cancer treated with curative intent: results from the QUASAR 2 clinical trial and an Australian community-based series

**DOI:** 10.1016/S2468-1253(18)30117-1

**Published:** 2018-07-02

**Authors:** Enric Domingo, Carme Camps, Pamela J Kaisaki, Marie J Parsons, Dmitri Mouradov, Melissa M Pentony, Seiko Makino, Michelle Palmieri, Robyn L Ward, Nicholas J Hawkins, Peter Gibbs, Hanne Askautrud, Dahmane Oukrif, Haitao Wang, Joe Wood, Evie Tomlinson, Yasmine Bark, Kulvinder Kaur, Elaine C Johnstone, Claire Palles, David N Church, Marco Novelli, Havard E Danielsen, Jon Sherlock, David Kerr, Rachel Kerr, Oliver Sieber, Jenny C Taylor, Ian Tomlinson

**Affiliations:** a Oxford Centre for Cancer Gene Research, Wellcome Trust Centre for Human Genetics, Oxford, UK; b Genomic Medicine Theme, National Institute for Health Research Oxford Biomedical Research Centre, Wellcome Trust Centre for Human Genetics, Oxford, UK; c Department of Oncology, University of Oxford, Oxford, UK; d Systems Biology and Personalised Medicine Division, Walter and Eliza Hall Institute of Medial Research, Parkville, VIC, Australia; e Department of Surgery, University of Melbourne, Parkville, VIC, Australia; f Department of Medical Biology, University of Melbourne, Parkville, VIC, Australia; g Office of the Deputy Vice-Chancellor (Research), University of Queensland, Brisbane, QLD, Australia; h Faculty of Medicine, University of Queensland, Brisbane, QLD, Australia; i Department of Medical Oncology, Royal Melbourne Hospital, Parkville, VIC, Australia; j Institute for Cancer Genetics and Informatics, Oslo University Hospital, Oslo, Norway; k Department of Histopathology, University College London, London, UK; l Thermo Fisher Scientific, Paisley, UK; m Nuffield Department of Clinical and Laboratory Science, Radcliffe Department of Medicine, John Radcliffe Hospital, Oxford, UK; n School of Biomedical Sciences, Monash University, Clayton, VIC, Australia; o Cancer Genetics and Evolution Laboratory, Institute of Cancer and Genomic Sciences, University of Birmingham, Edgbaston, Birmingham, UK

## Abstract

**Background:**

Molecular indicators of colorectal cancer prognosis have been assessed in several studies, but most analyses have been restricted to a handful of markers. We aimed to identify prognostic biomarkers for colorectal cancer by sequencing panels of multiple driver genes.

**Methods:**

In stage II or III colorectal cancers from the QUASAR 2 open-label randomised phase 3 clinical trial and an Australian community-based series, we used targeted next-generation sequencing of 82 and 113 genes, respectively, including the main colorectal cancer drivers. We investigated molecular pathways of tumorigenesis, and analysed individual driver gene mutations, combinations of mutations, or global measures such as microsatellite instability (MSI) and mutation burden (total number of non-synonymous mutations and coding indels) for associations with relapse-free survival in univariable and multivariable models, principally Cox proportional hazards models.

**Findings:**

In QUASAR 2 (511 tumours), *TP53, KRAS, BRAF*, and *GNAS* mutations were independently associated with shorter relapse-free survival (p<0·035 in all cases), and total somatic mutation burden with longer survival (hazard ratio [HR] 0·81 [95% CI 0·68–0·96]; p=0·014). MSI was not independently associated with survival (HR 1·12 [95% CI 0·57–2·19]; p=0·75). We successfully validated these associations in the Australian sample set (296 tumours). In a combined analysis of both the QUASAR 2 and the Australian sample sets, mutation burden was also associated with longer survival (HR 0·84 [95% CI 0·74–0·94]; p=0·004) after exclusion of MSI-positive and *POLE* mutant tumours. In an extended analysis of 1732 QUASAR 2 and Australian colorectal cancers for which *KRAS, BRAF*, and MSI status were available, *KRAS* and *BRAF* mutations were specifically associated with poor prognosis in MSI-negative cancers. MSI-positive cancers with *KRAS* or *BRAF* mutations had better prognosis than MSI-negative cancers that were wild-type for *KRAS* or *BRAF*. Mutations in the genes *NF1* and *NRAS* from the MAPK pathway co-occurred, and mutations in the DNA damage-response genes *TP53* and *ATM* were mutually exclusive. We compared a prognostic model based on the gold standard of clinicopathological variables and MSI with our new model incorporating clinicopathological variables, mutation burden, and driver mutations in *KRAS, BRAF*, and *TP53*. In both QUASAR 2 and the Australian cohort, our new model was significantly better (p=0·00004 and p=0·0057, respectively, based on a likelihood ratio test).

**Interpretation:**

Multigene panels identified two previously unreported prognostic associations in colorectal cancer involving *TP53* mutation and total mutation burden, and confirmed associations with *KRAS* and *BRAF*. Even a modest-sized gene panel can provide important information for use in clinical practice and outperform MSI-based prognostic models.

**Funding:**

UK Technology Strategy Board, National Institute for Health Research Oxford Biomedical Research Centre, Cancer Australia Project, Cancer Council Victoria, Ludwig Institute for Cancer Research, Victorian Government.

## Introduction

There is increasing recognition that treatment of common cancers can be modified according to a patient's expected prognosis or response to therapy. For some new molecularly guided therapies, powerful biomarkers of response are available, which often comprise mutations in the specific protein that is targeted. However, for conventional cytotoxic therapies, predictive markers of response are rare. In view of the modest survival benefits that conventional cytotoxic therapies provide for patients with common solid malignancies, biomarkers of prognosis still have substantial potential clinical importance. Such markers could guide the use of more or less aggressive treatment regimens and enable clinicians to balance expected outcomes against early and late therapeutic toxicities.

**Research in context**

**Evidence before this study**

The decision to give adjuvant chemotherapy after resection of stage II or III colorectal cancer is based mainly on pathological factors such as tumour and nodal stage. Microsatellite instability (MSI) is the only molecular marker used routinely in this setting. However, patient outcomes remain variable, and stratification needs to be improved. We searched PubMed with the terms “prognosis”, “colorectal”, “colon”, and “rectal” for articles published in English up to Feb 16, 2017. Only two large studies (>400 profiled patients) in the adjuvant setting had screened more than four molecular markers.

**Added value of this study**

We used next-generation sequencing to analyse a panel of 82 genes in colorectal cancer from the QUASAR 2 clinical trial, and validated our findings in an Australian community-based colorectal cancer cohort. We identified high mutation burden as an independent marker of good prognosis, even after omitting hypermutant tumours with defects in DNA mismatch or polymerase proofreading repair. We hypothesise that this finding resulted from high neo-epitope levels genome-wide. *TP53, KRAS*, and *BRAF* mutations were additionally independently associated with poor prognosis, although the association with *BRAF* and *KRAS* was restricted to MSI-negative tumours.

**Implications of all the available evidence**

Although the 15% of stage II or III colorectal cancers with hypermutation caused by DNA repair defects have previously been shown to have a good prognosis in the non-metastatic setting, we have shown that increased mutation burden among non-hypermutated colorectal cancers is also associated with favourable outcomes. Our data additionally show that the prognostic value of MSI is improved by a model based on mutation burden and *KRAS, BRAF*, and *TP53* mutations. Use of even a modestly sized gene panel provides superior prognostic information to tests based on a handful of genes, and could allow for existing and novel therapies to be targeted to subgroups of patients with poor prognosis, thereby sparing patients with good outcomes unnecessary and toxic treatment.

Biomarkers can be based on several different types of molecule, and high-profile work has highlighted the potential use of mRNA profiling for identification of groups of colorectal cancers with varying prognoses.^1^ Other biomarkers are based on DNA, which is more stable and thus generally easier to analyse than mRNA. For colorectal cancers treated with curative intent, the biomarker most consistently used in clinical practice is microsatellite instability (MSI), which usually results from defective DNA mismatch repair.^2^ For stage II colorectal cancers, MSI predicts good recurrence-free survival, with hazard ratios (HRs) as low as 0·6.^3^, ^4^ This association is less strong for stage III cancers, and, in stage IV colorectal cancers, MSI positivity is probably associated with poor prognosis.^5^

The availability of a few large datasets (>500 participants) from clinical trials has begun to clarify the associations between some somatic mutations and prognosis of colorectal cancers. However, most of these analyses have been restricted to *KRAS* mutations, *BRAF* mutations, or MSI (appendix pp 6–7). Overall, for colorectal cancers treated with curative intent (generally stage II or III), data support an association between MSI and good prognosis, and weaker evidence suggests that *KRAS* and *BRAF* mutations, which are mutually exclusive, indicate poor prognosis in MSI-negative tumours.^6^, ^7^, ^8^, ^9^, ^10^, ^11^ However, MSI-positive colorectal cancers tend to be *BRAF*-mutant and *KRAS*-wild-type, so statistical interactions could exist between these prognostic biomarkers. Furthermore, whether combinations of other genetic biomarkers provide useful prognostic information is unclear.

Screening has been restricted to only a few genes in large genetic biomarker studies for two main reasons: suboptimal sample quality or quantity, and the cost of mutation screening. Because somatic mutations tend to co-occur in molecular pathways of tumorigenesis, screening of many potentially prognostic mutations in the same dataset would be highly desirable to identify the primary determinants of tumour behaviour. However, the few studies in which such analyses were done did not have standardised recruitment and follow-up. The prime example is the exome or genome sequencing of over 600 colorectal cancers by the Cancer Genome Atlas group.^12^ This work provided an excellent dataset for discovery of driver mutations, but is of little use for biomarker discovery owing to the heterogeneity of the sample set and associated variability in clinical data.

In this exploratory study, we aimed to retain the advantages of a large clinical trial dataset while assessing several prognostic biomarkers for colorectal cancers. To this end, we used an 82-gene panel to identify somatic mutations in all the major colorectal cancer driver genes in more than 500 tumours from the QUASAR 2 clinical trial of stage II and III colorectal cancers. We also assessed MSI and the ultramutator phenotype resulting from *POLE* mutations.^4^ We also tested a larger QUASAR 2 sample set for *KRAS* and *BRAF* mutations and MSI. Variables associated with survival in QUASAR 2 were replication tested in an independent community-based cohort, and subjected to a combined analysis.

## Methods

### Study design and participants

In this exploratory study, we assessed prognostic biomarkers for colorectal cancers in a large clinical trial dataset from a phase 3 clinical trial (QUASAR 2) and an independent community-based validation cohort. QUASAR 2 was an open-label, randomised phase 3 clinical trial^13^ comprising 1952 patients with high-risk stage II or stage III colorectal cancer, who were randomly assigned to capecitabine alone or capecitabine plus bevacizumab, without radiotherapy. Median follow-up was 4·92 years (IQR 4·00–5·16). Overall or disease-free survival did not differ significantly between the two groups at 3 years' follow-up.^13^ Similar results have been recorded in two other trials.^14^, ^15^ We obtained clinicopathological data (appendix p 8) from the QUASAR 2 trial database. Some data were converted to binary variables—ie, sex, location (proximal *vs* distal), and depth of invasion (T4 *vs* T1, T2, or T3) and lymph node metastasis (N2 or N1 *vs* N0) according to the TNM grading system. Age and grade were assessed as continuous variables.

The community-based series included 657 patients with stage II or III colorectal cancer who were treated at the Royal Melbourne Hospital (Parkville, VIC, Australia), Western Hospital Footscray (Footscray, VIC, Australia) or St Vincent's Hospital (Sydney, NSW, Australia) between Jan 1, 1993, and Dec 31, 2009 (appendix p 8). Individuals with hereditary colorectal cancer syndromes were excluded. All patients received standard neoadjuvant or adjuvant fluorouracil-based chemotherapy or concurrent chemoradiotherapy. In this patient series, stage II disease was deemed low risk when tumours were T3/N0; otherwise it was judged high risk. All patients provided written informed consent, and the study was approved by medical ethics committees at all three sites.

### Procedures

Colorectal cancer samples from UK QUASAR 2 were collected for molecular analysis. 40 μm scrolls were cut from formalin-fixed paraffin-embedded specimens of colorectal cancers that had greater than 80% estimated purity, and from healthy bowel; 10 μm sections were cut from the remaining colorectal cancers and needle microdissected to enrich for tumours with a haematoxylin and eosin section as a guide. Peripheral blood samples were also available from most patients. DNA was extracted from formalin-fixed paraffin-embedded tissue with the DNeasy kit (Qiagen, Hilden, Germany) and from blood with the Maxwell 16 Blood DNA Purification Kit (Promega, Madison, WI, USA). The whole cohort was analysed by Sanger sequencing for selected mutations and for MSI (appendix pp 1–3), and a subset of tumours was also analysed with an Ion Torrent (Life Technologies, Guildorf, CT, USA) sequencing gene panel for 82 genes (appendix p 9). We eliminated mutations with a high probability of being artifacts and cancers with high levels of artifactual hypermutation owing to ex-vivo cytosine deamination (appendix pp 2–3, 32).

We identified all probable driver mutations (appendix p 14) and selected the 13 most commonly mutated genes (ie, mutated in eight or more tumours) for further analysis to identify mutations tending to occur together in genetic pathways (appendix pp 17, 35–36). High-depth sequencing allowed us to identify tumours carrying somatic mutations at substantially reduced allele frequency (suggestive of subclonal status).

From the community-based series, fresh-frozen tumours and matched normal specimens were retrieved from hospital tissue banks. A subset of these tumours was screened in 113 genes by targeted next-generation sequencing, the others were screened with conventional PCR-based sequencing (appendix pp 4–5); choice of screening method was based on the availability of funding. All patients were prospectively followed up per the standard Australian National Health and Medical Research Council guidelines, with a median follow-up of 60 months (IQR 36–69).

We investigated the prognostic associations of *KRAS* and *BRAF* mutations in relation to MSI status by pooling data from the QUASAR 2 gene panel, the Australian validation set, and additional QUASAR 2 and stage II or III Australian colorectal cancers that had been analysed for MSI and by Sanger sequencing for *KRAS* or *BRAF* mutations (appendix p 8) for an extended set of patients. Similar analyses were also done in the extended cohorts, whereby *TP53* status derived from either next-generation sequencing or Sanger sequencing was added.

### Statistical analysis

Individual driver gene mutations, combinations of mutations, or global measures such as MSI or mutation burden (total number of non-synonymous mutations and coding indels) were tested for associations with relapse-free survival in univariable and multivariable models, principally Cox proportional hazards models in accordance with published guidelines (appendix p 10).^16^ We used the likelihood ratio test to compare a prognostic model based on the gold standard of clinicopathological variables and MSI with our new model, and did 10% leave-out cross-validation analysis to confirm the robustness of these results. To test whether the prognostic effect of mutation burden was due to hypermutation only, the same model was run in the subset of tumours without MSI or pathogenic *POLE* mutations. All survival analyses were two-sided and were deemed significant if p values were less than or equal to 0·05. Univariable results with p values less than 0·1 were taken forward to be tested in multivariable models. Further details of patients and analytic methods are in the appendix (p 5).

Because several mutations co-varied, we searched for primary associations by multivariable regression, hierarchical clustering, and Bayesian networks (appendix p 4). All analyses were done in STATA (version 10), R (version 3.4.1), or Banjo (version 2.2.0). Research materials supporting this publication can be accessed by contacting the corresponding author.

### Role of the funding source

The study funders had no role in the study design; data collection, analysis, or interpretation; or writing of the report. The corresponding author had full access to all study data and final responsibility for the decision to submit for publication.

## Results

598 tumours from the QUASAR 2 clinical trial were sequenced for 82 genes. After exclusion of mutations with a high probability of being artifacts and cancers with high levels of artifactual hypermutation owing to ex-vivo cytosine deamination, 511 tumours remained for further analysis (appendix pp 2–3).

The 13 most commonly mutated genes (*APC, TP53, KRAS, PIK3CA, BRAF, FBXW7, SMAD4, ATM, PTEN, NF1, CTNNB1, GNAS*, and *NRAS*)—ie, mutated in eight or more tumours—were selected for further analysis to identify mutations tending to occur together in genetic pathways (appendix p 14). In addition to known associations, such as those between *BRAF* mutation and MSI or between mutations of *KRAS* and *PIK3CA*, new unreported ones were found. Multivariable regression, hierarchical clustering, and Bayesian networks showed that mutations in *NF1*, a negative regulator of the Ras pathway, were positively associated with *NRAS* mutations, but not with mutations in *KRAS* or *BRAF* (appendix pp 17, 35–36). *SMAD4* mutations were associated with *BRAF* mutations but not with *KRAS* or *NRAS* changes (appendix pp 17, 35–36), suggesting possible synergy between *BRAF* and the TGFβ or BMP pathways. Additionally, logistic regression and Bayesian network analyses showed a strong negative association between driver mutations in *TP53* and *ATM* (appendix pp 17, 35–36). Clustering and Bayesian network analysis suggested a positive association between *ATM* and *PTEN* mutations (appendix pp 17, 35–36). Regression analysis between molecular and clinical variables showed that *KRAS* mutations were associated with female sex (similar to *BRAF* mutations;^12^, ^17^ appendix pp 17, 35–36). Additionally, mutations in *FBXW7* and *CTNNB1* were associated with high-grade disease (appendix pp 17, 35–36).

High-depth sequencing identified 58 (11%) tumours carrying somatic mutations at substantially reduced allele frequency, suggesting subclonal status. Of the 13 most commonly mutated genes, *PIK3CA* (p=0·001), *ATM* (p=0·002), and SMAD4 (p=0·05) had lower driver mutation allele frequencies than the other genes, suggesting they were more often subclonal (appendix p 18). Mutation burden, clonal diversity (presence of any identified mutation at low allele frequency), and driver mutations in the 13 genes were tested for prediction of bevacizumab treatment response, with no significant associations identified (data not shown).

In QUASAR 2, overall mutation burden and mutations in four specific genes (*TP53, KRAS, BRAF*, and *GNAS*) showed promising individual associations with relapse-free survival (predefined p<0·10) and were thus selected for multivariable analysis, together with T stage, N stage, treatment group (because bevacizumab had previously been associated with poor prognosis in our patient subgroup, although not the whole trial), and MSI (which co-varied with mutation burden and is probably the best established prognostic factor for colorectal cancer; table 1, appendix p 19). Mutation burden (HR 0·81 [95% CI 0·68–0·96]; p=0·014), mutations in *TP53, KRAS, BRAF*, and *GNAS*, T stage, N stage, and use of bevacizumab were all independently associated with poor prognosis (ie, p≤0·05), but MSI was not (HR 1·12 [95% CI 0·57–2·19]; p=0·75; table 1). To test whether the prognostic effect of mutation burden was due to hypermutation only, the same model was run in the subset of tumours without MSI or pathogenic *POLE* mutations. Mutation burden was no longer significantly associated with outcome (HR 0·85 [95% CI 0·73–1·00]; p=0·051), although the HR was similar. The other variables retained significance similar to that previously shown (table 1).

**Table 1:** Associations between clinicopathological molecular variables and relapse-free survival in the QUASAR 2 cohort

	**All cases univariable (n=511)**			**All cases multivariable (n=511)**			**MSI-negative and non-pathogenic *POLE* multivariable (n=443)**		
	HR	95% CI	p value	HR	95% CI	p value	HR	95% CI	p value
*KRAS* mutation	1·48	1·07–2·05	0·018	1·99	1·37–2·91	3·44 × 10^−4^	2·25	1·51–3·35	6·07 × 10^−5^
*BRAF* mutation	1·42	0·94–2·13	0·093	2·46	1·51–4·03	3·31 × 10^−4^	2·88	1·70–4·85	7·50 × 10^−5^
*TP53* mutation	1·53	1·08–2·18	0·018	1·63	1·12–2·38	0·011	1·61	1·09–2·38	0·025
*GNAS* mutation	2·19	0·89–5·35	0·087	2·76	1·08–7·04	0·034	4·00	1·42–11·3	0·009
Mutation burden (quartiles)	0·87	0·75–1·00	0·055	0·81	0·68–0·96	0·014	0·85	0·73–1·00	0·051
MSI	0·73	0·42–1·28	0·271	1·12	0·57–2·19	0·75	..	..	..
Chemotherapy (bevacizumab plus capecitabine *vs* capecitabine)	1·37	0·98–1·92	0·065	1·43	1·02–2·00	0·039	1·55	1·09–2·22	0·015
T4 *vs* T1, T2, or T3*	2·11	1·52–2·94	8·59 × 10^−6^	2·10	1·50–2·93	1·36 × 10^−5^	2·29	1·61–3·25	3·66 × 10^−6^
N1 or N2 *vs* N0*	1·80	1·22–2·63	0·003	1·85	1·25–2·73	0·002	2·03	1·33–3·09	0·001

Cox proportional hazards analysis was done. The univariable analyses were adjusted by T stage, N stage, and treatment arm (or two of these if the adjustment variable itself was being assessed). Multivariable analysis was based on all variables shown. Mutation burden was derived from total number of non-synonymous mutations and coding indels, which are most likely to be functionally relevant, but similar results were obtained when other somatic variants were also included (appendix). *POLE* proofreading mutation is not shown as a prognostic variable because of the low frequency of those cancers (appendix). MSI=microsatellite instability. HR=hazard ratio.

* According to TNM tumour classification.

In the Australian community-based cohort, 379 patients received adjuvant fluorouracil treatment, of whom 47 also received oxaliplatin (no data for oxaliplatin use were available for 38). We replication tested our prognostic markers in 296 tumours from the Australian cohort (appendix pp 8, 37–38), in which all prognostic markers identified in QUASAR 2 (except *GNAS* mutations) had been assessed. A multivariable analysis incorporating the same clinical and molecular variables and co-variables showed that, in agreement with the QUASAR 2 analysis, *BRAF* mutation, *TP53* mutation, and mutation burden were associated (p≤0·05) with relapse-free survival, whereas MSI was not (table 2). *KRAS* mutation also showed a similar prognostic association in the Australian patients to that present in QUASAR 2, but this was not statistically significant. When MSI-positive and ultramutator tumours were excluded from the Australian analysis*, KRAS* mutation was significantly associated with prognosis, but *BRAF* mutation was not (table 2).

**Table 2:** Associations between clinicopathological molecular variables and relapse-free survival in the Australian community-based series

	All cases univariable (n=296)*			**All cases multivariable (n=253)**			**MSI negative and non-pathogenic *POLE* multivariable (n=209)**		
	HR	95% CI	p value	HR	95% CI	p value	HR	95% CI	p value
*KRAS* mutation	1·31	0·92–1·87	0·136	1·51	0·97–2·38	0·066	1·61	1·02–2·59	0·040
*BRAF* mutation	0·91	0·52–1·64	0·780	2·18	1·08–4·56	0·029	1·79	0·73–4·24	0·204
*TP53* mutation	1·19	0·83–1·71	0·334	1·82	1·12–2·73	0·014	1·81	1·09–2·82	0·020
Mutation burden (quartiles)	0·72	0·62–0·85	8·62 × 10^−5^	0·78	0·63–0·95	0·014	0·82	0·64–0·93	0·008
MSI	0·39	0·18–0·71	0·003	0·62	0·24–1·44	0·247	..	..	..
Chemotherapy (yes *vs* no)	1·01	0·71–1·44	0·946	0·60	0·34–0·91	0·019	0·51	0·18–0·90	0·018
Radiotherapy (yes *vs* no)	1·21	0·50–3·02	0·653	1·33	0·53–3·32	0·546	1·29	0·51–3·20	0·603
T4 *vs* T1, T2, or T3†	2·19	1·54–3·22	2·01 × 10^−5^	2·38	1·57–3·75	6·34 × 10^−5^	2·67	1·73–4·21	1·62 × 10^−5^
N1 or N2 *vs* N0†	1·40	0·97–2·08	0·070	1·21	0·71–2·04	0·493	1·19	0·66–2·05	0·597

Cox proportional hazards analysis was done. The univariable analyses were adjusted by T stage, N stage, and treatment group (or two of these if the adjustment variable itself was being assessed). Multivariable analysis was based on all variables shown. Mutation burden was derived from total number of non-synonymous mutations and coding indels, which are most likely to be functionally relevant, but similar results were obtained when other somatic variants were also included (appendix). *POLE* proofreading mutation is not shown as a prognostic variable because of the low frequency of those cancers (appendix). *BRAF* was tested only for the common V600E variant. *GNAS* was not tested. MSI=microsatellite instability. HR=hazard ratio.

* Missing data for *KRAS* (n=9), *BRAF* (n=11), *TP53* (n=10), mutation burden (n=11), MSI (n=1), and radiotherapy (n=21).

† According to TNM tumour classification.

**Figure 1:**
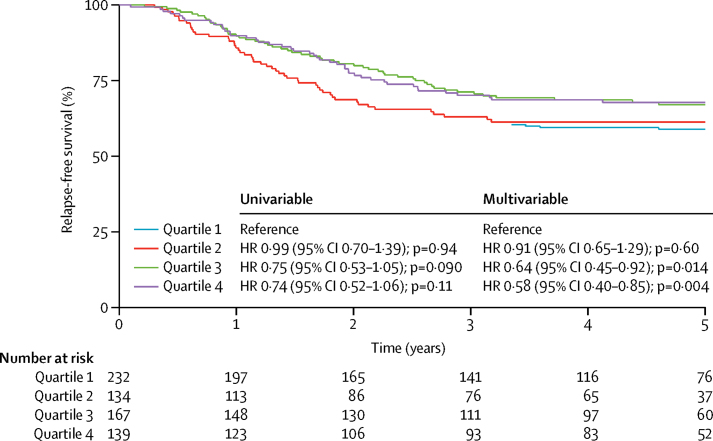
Relapse-free survival in the combined QUASAR 2 and Australian cohorts by mutation burden from gene-panel analysis (n=672) Burden data are shown by quartile (highest burden in quartile 4). Cancers that were positive for microsatellite instability or with pathogenic *POLE* mutations were excluded. Cox proportional hazards model results are shown for univariable and multivariable analyses with quartile 1–4 as a continuous variable and other co-variables as per table 3. The numbers in each quartile are not equal because of ties in mutation burden. HR=hazard ratio.

A combined analysis of the QUASAR 2 and Australian cohorts (n=807), showed that mutations in *KRAS, BRAF*, and *TP53*, and lower mutation burden were all independently associated with poor prognosis, whereas MSI was not (figure 1; table 3; appendix p 20). Exclusion of MSI-positive and ultramutator cancers did not affect our findings (table 3). No significant heterogeneity was noted between cohorts and our model persisted in Australian patients treated with chemotherapy (data not shown).

**Table 3:** Associations between clinicopathological molecular variables and relapse-free survival in the combined QUASAR 2 and Australian community-based series population

	All cases univariable (n=807)*			**All cases multivariable (n=764)**			**MSI-negative and non-pathogenic *POLE* multivariable (n=652)**		
	HR	95% CI	p value	HR	95% CI	p value	HR	95% CI	p value
*KRAS* mutation	1·40	1·10–1·78	0·006	1·74	1·31–2·29	1·21 × 10^−4^	1·88	1·40–2·51	2·11 × 10^−5^
*BRAF* mutation	1·23	0·88–1·72	0·231	2·21	1·47–3·29	1·02 × 10^−4^	2·32	1·50–3·58	1·49 × 10^−4^
*TP53* mutation	1·30	1·01–1·67	0·039	1·65	1·24–2·19	4·67 × 10^−4^	1·68	1·24–2·26	0·001
Mutation burden (quartiles)	0·82	0·74–0·92	5·1 × 10^−4^	0·8	0·70–0·91	0·001	0·84	0·74–0·94	0·004
MSI	0·58	0·38–0·89	0·012	0·8	0·46–1·35	0·399	..	..	..
Cohort plus treatment QUASAR 2 capecitabine	Reference	..	..	Reference	..	..	Reference	..	..
Cohort plus treatment QUASAR 2 bevacizumab plus capecitabine	1·45	1·04–2·03	0·029	1·44	1·02–2·01	0·034	1·53	1·07–2·18	0·019
Cohort plus treatment Australia no chemotherapy	2·04	1·4–2·98	2·2 × 10^−4^	3·48	2·28–5·30	7·04 × 10^−9^	4·05	2·58–6·34	9·96 × 10^−10^
Cohort plus treatment Australia chemotherapy	2·06	1·45–2·93	5·61 × 10^−6^	1·75	1·18–2·58	0·005	1·88	1·25–2·83	0·002
Radiotherapy (yes *vs* no)	1·56	0·64–3·78	0·326	1·37	0·54–3·41	0·503	1·3	0·51–3·24	0·579
T4 *vs* T1, T2, or T3†	1·81	1·42–2·29	1·30 × 10^−6^	2·19	1·68–2·83	3·03 × 10^−9^	2·36	1·80–3·09	4·38 × 10^−10^
N1 or N2 *vs* N0†	1·45	1·11–1·89	0·006	1·63	1·21–2·20	0·001	1·68	1·21–2·30	0·002

Cox proportional hazards analysis was done. The univariable analyses were adjusted by T stage, N stage, and treatment group (or two of these if the adjustment variable itself was being assessed). Multivariable analysis was based on all variables shown. Mutation burden was derived from total number of non-synonymous mutations and coding indels, which are most likely to be functionally relevant, but similar results were obtained when other somatic variants were also included (appendix). *POLE* proofreading mutation is not shown as a prognostic variable because of the low frequency of those cancers (appendix). Mutation burden quartile was derived separately for the QUASAR 2 and Australian cohorts because of the different content of the two panels. The cohort/treatment variables are categorical. MSI=microsatellite instability. HR=hazard ratio.

* Missing data from Australian cohort for *KRAS* (n=9), *BRAF* (n=11), *TP53* (n=10), mutation burden (n=11), MSI (n=1), and radiotherapy (n=21).

† According to TNM tumour classification.

We compared a prognostic model based on the gold standard of clinicopathological variables and MSI with our new model incorporating clinical variables, mutation burden, and driver mutations in *KRAS, BRAF*, and *TP53.* In both QUASAR 2 and the Australian cohort, our new model was significantly better (p=0·00004 and p=0·0057, respectively, based on the likelihood ratio test). A 10% leave-out cross-validation analysis showed these analyses to be robust (appendix p 5).

We explored the prognostic model separately in stage II (n=266) and stage III (n=499) colorectal cancers and found that the model was significant (p=7·3 × 10^−8^) only in stage III disease (appendix pp 21–22), but HRs were similar in both stages. Correspondingly, despite inherently reduced power, an analysis by tumour location (proximal colon, distal colon, rectum) showed similar HRs for all biomarkers across sites, even after exclusion of hypermutated tumours (appendix pp 23–25). Additionally, formal assessment of interactions between individual biomarkers and stage or tumour location showed no evidence of significant deviation from a log-additive model (data not shown).

On the basis of previous reports,^6^, ^7^, ^8^, ^9^, ^10^, ^11^ we investigated the prognostic associations of *KRAS* and *BRAF* mutations in relation to MSI status by pooling data from an extended set of the QUASAR 2 and Australian cohorts, including an additional 676 colorectal cancers from QUASAR 2 and 362 stage II or III colorectal cancers from the Australian cohort (n=1732). In multivariable analysis, MSI was associated with good prognosis (HR 0·45 [95% CI 0·31–0·64]; p=0·00001), and *KRAS* (1·22 [1·01–1·48]; p=0·035] and *BRAF* (1·53 [1·14–2·04]; p=0·004) mutations were both associated with poor prognosis (appendix p 26). Because the strong co-variation of these biomarkers could have confounded or obscured prognostic effects, we added multiplicative interaction terms between MSI and mutations in *KRAS* and *BRAF* to the multivariable model. Both of these interactions were significant (p=0·003 and p=0·023, respectively), suggesting differential prognostic effects.

**Figure 2:**
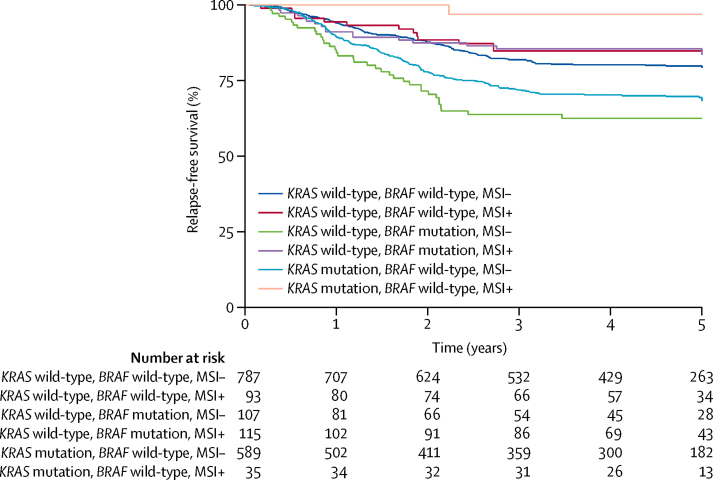
Relapse-free survival by combinations of MSI and mutations in *KRAS* and *BRAF* in the combined extended QUASAR 2 and Australian cohorts Cancers with pathogenic *POLE* mutations were excluded. MSI=microsatellite instability.

Accordingly, we explored different combinations of MSI, *KRAS* mutation, and *BRAF* mutation. Compared with triple-negative (ie, MSI-negative, *KRAS* and *BRAF* wild-type) cancers, MSI-negative tumours with *KRAS* (HR 1·35 [95% CI 1·11–1·64]; p=0·003) or *BRAF* (2·02 [1·47–2·76]; 1·20 × 10^−5^) mutations were associated with worse prognosis (table 4, figure 2). By contrast, MSI-positive colorectal cancers with *KRAS* (HR 0·28 [95% CI 0·09–0·89]; p=0·028) or *BRAF* (0·55 [0·35–0·90]; p=0·017) mutations were associated with a significantly better prognosis than the triple negatives (table 4), although the difference was not significant compared with MSI-positive colorectal cancers without *KRAS* or *BRAF* mutations. The six main subgroups combining *MSI, KRAS*, and *BRAF* had consistent effects between the QUASAR 2 and Australian cohorts (data not shown).

**Table 4:** Prognosis associated with subgroups by *KRAS* mutation, V600E *BRAF* mutation, and MSI in all cohorts (n=1732)

	**Hazard ratio**	**95% CI**	**p value**
*KRAS* wild-type, *BRAF* wild-type, MSI negative	Reference	..	..
*KRAS* mutated, *BRAF* wild-type, MSI negative	1·35	1·11–1·64	0·003
*KRAS* wild-type, *BRAF* mutated, MSI negative	2·02	1·47–2·76	1·20 × 10^−5^
*KRAS* wild-type, *BRAF* wild-type, MSI positive	0·90	0·56–1·45	0·670
*KRAS* mutated, *BRAF* wild-type, MSI positive	0·28	0·09–0·89	0·028
*KRAS* wild-type, *BRAF* mutated, MSI positive	0·55	0·35–0·90	0·017
T4 *vs* T1, T2, or T3*	2·26	1·88–2·71	3·32 × 10^−18^
N1 or N2 *vs* N0*	2·07	1·65–2·59	2·62 × 10^−10^

The p value for the interaction between MSI and *BRAF* is 0·003; the p value for the interaction between MSI and *KRAS* is 0·023. Results are from multivariable analysis adjusted by cohort groups. Six patients in very rare subgroups are not shown. MSI=microsatellite instability.

* According to TNM tumour classification.

**Figure 3:**
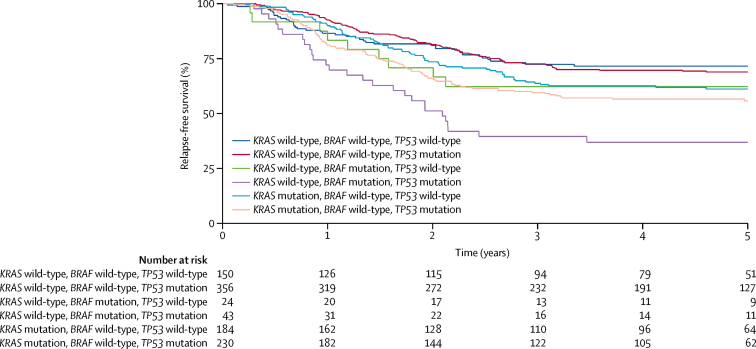
Relapse-free survival by combinations of mutations in *KRAS, BRAF*, and *TP53* in MSI-negative tumours in the combined extended QUASAR 2 and Australian cohorts Cancers that were MSI positive or with pathogenic *POLE* mutations were excluded. MSI=microsatellite instability.

Although MSI was not an independent prognostic marker when mutation burden was also assessed, it was prognostic in the absence of information about mutation burden (appendix p 26). We therefore explored whether new prognostic groups within the larger MSI-negative subset could be identified with *KRAS, BRAF*, and *TP53*, given that *TP53* mutation remained an independent prognostic marker when MSI-positive and ultramutator colorectal cancers were excluded from the main analysis based on gene panels (table 1). Within the MSI-negative colorectal cancer set (n=991), tumours with *BRAF* and *TP53* mutations had a particularly poor prognosis (HR 3·08 [95% CI 1·88–5·03]; p=7·12 × 10^−6^; figure 3; appendix p 27). Neither the interaction between *TP53* and *BRAF* (HR 2·21 [95% CI 0·97–5·03]; p=0·058), nor that between *TP5*3 and *KRAS* (1·13 [0·71–1·80]; p=0·62) were significant.

## Discussion

In this study, we used overlapping cancer gene mutation panels to analyse a cohort from a high-quality clinical trial of colorectal cancers treated with curative intent and a validation cohort. In multivariable analysis incorporating known clinicopathological prognostic factors, we showed that low overall mutation burden and mutations in *KRAS, BRAF*, and *TP53* were independently associated with decreased relapse-free survival after colorectal cancer treated with curative intent. These findings were present both in the clinical trial cohort and in the Australian validation set of community-based patients. The fact that we found no molecular marker for bevacizumab response in QUASAR 2 or chemotherapy response in the Australian cohorts suggests that the markers we identified are prognostic, although formal demonstration of this hypothesis is difficult because most patients received fluorouracil-based chemotherapy.

Use of prognostic molecular markers in management of solid tumours is still not widespread, partly because of a lack of validated markers and partly because of differences between studies, leading to uncertainty about which markers to use and their estimated effect sizes. Although molecular indicators of colorectal cancer prognosis have been assessed in several large studies, analyses in most cases have been restricted to a handful of markers.

The complexity of associations between mutations and colorectal cancer prognosis is arguably reflected by the generally stronger associations of markers in our multivariable than in univariable analyses. Furthermore, MSI was generally not prognostic in our analyses, because its effects were captured by mutation burden (somatic single nucleotide variants and small indels). However, mutation burden not only strongly co-varied with MSI and *POLE,* but also provided prognostic information in MSI-negative colorectal cancers. Although high mutation burden has been associated with good colorectal cancer prognosis in the context of MSI and *POLE* proofreading deficiency,^4^ this relation has not previously been shown for colorectal cancers without those forms of genomic instability. Similar data for other tumour types are few,^18^, ^19^, ^20^ although in other cancers with generally high mutation burdens but without specific forms of genomic instability, such as lung carcinoma and melanoma, mutation burden has predicted response to immune checkpoint inhibitors.^21^, ^22^

In our study, undetected hypermutator or ultramutator cancers could have contributed to the mutation burden association, although the frequencies of MSI and *POLE* mutations that we recorded were typical of other studies,^4^ and we identified a monotonic relationship between mutation burden quartile and relapse-free survival. Another potential cause of the mutation burden association was non-excluded deamination artifacts if they happened to be associated with an unknown factor correlated with good prognosis. However, we made strenuous efforts to exclude those artifacts, no plausible explanatory causes such as tumour age were detectable within QUASAR 2, and the Australian validation cohort analyses were done in fresh frozen tissue, which was unlikely to have deamination. In our study, the association between prognosis and mutation burden was sufficiently strong that even a modestly sized gene panel should pick it up, suggesting that it was representative of mutation burden in the exome.^23^ The underlying reason for that association is unclear, although anti-tumour immune responses are evidently the prime candidate.^18^, ^19^, ^20^

We showed a strong negative association between driver mutations in *TP53* and *ATM*, two key mediators in the DNA damage response, suggesting that these mutations are alternative DNA damage response inactivators. We also found a positive association between *ATM* and *PTEN* mutations; PTEN is phosphorylated by ATM in response to DNA-damaging agents, thus inducing autophagy.^24^ Mutations in *FBXW7* and *CTNNB1* were associated with high-grade disease, the latter suggesting that activation of the Wnt pathway through *CTNNB1* rather than *APC* mutation might predispose to poorly differentiated colorectal cancers.

The interplay between *KRAS, BRAF*, and *TP53* mutations, MSI, and mutation burden in our data set is intriguing. These mutations co-vary strongly (appendix), and are additionally associated with other molecular variables. Thus, to decipher primary associations is extremely challenging. Nevertheless, our study strongly supports the reported poor prognosis of MSI-negative colorectal cancers with *KRAS* or *BRAF* mutations^6^, ^7^, ^8^, ^9^, ^10^, ^11^ compared with MSI-negative colorectal cancers wild-type for these genes and unselected MSI-positive colorectal cancers. Additionally, we showed that *KRAS* or *BRAF* mutation could be associated with improved prognosis in MSI-positive colorectal cancers. *TP53* has not previously been consistently reported as a prognostic marker for colorectal cancer in the curative setting, but very few large studies have included a sufficiently comprehensive molecular analysis of *KRAS, BRAF, TP53*, and MSI. Notably, addition of these four prognostic markers improved outcome prediction compared with current clinical guidelines based on MSI.

The strengths of our study are that several potential biomarkers were screened in a large, high-quality clinical trial and a community-based cohort. We have carefully done quality-control analysis to derive high-quality mutation calls. For mutation burden, the study is arguably limited by the size of the gene panels used, and a larger panel or exome and genome sequencing might detect even stronger associations with prognosis. Limitations include the low numbers of patients with stage II disease in the sample set, which means that the utility of our model in such patients remains formally unproven. Although we found our model to be significant only in stage III disease (appendix p 22), HRs were similar in both stages, suggesting that the lack of significance for stage II disease was the result of lower power in that set. Furthermore, we cannot formally distinguish between the model being prognostic or predictive for fluorouracil response. Another potential weakness is the different treatment regimens used in each cohort, although regimen was incorporated as a co-variable in the analyses. Finally, our study might have suboptimal power to draw firm conclusions about outcomes in small patient groups or subgroups, such as those with combinations of several molecular variables.

Advances in molecular testing hold considerable promise for the delivery of precision cancer medicine, but their clinical use to date has largely been limited to analysis of small numbers of actionable variants. In colorectal cancer, these include *KRAS* and *NRAS* mutation testing for prediction of resistance to anti-EGFR therapies,^25^ and MSI, which identifies stage II tumours with excellent prognosis^26^ and stage IV tumours likely to respond to immune checkpoint inhibition. Our findings show that the use of even a modest-sized gene panel can provide clinically useful information beyond individual driver mutations. Tumour mutation burden displaced MSI and *POLE* as a marker of prognosis in multivariable analysis, thus extending the group of colorectal cancers with good prognoses to include those with high mutation burden in the absence of a specific underlying mutator phenotype. Although we were unable to test whether mutational load is predictive for immunotherapy response, this correlation is well documented in other tumour types, including melanoma and lung and ovarian cancers.^27^ Accordingly, our results suggest that the use of tumour mutation burden as a prognostic and predictive marker in colorectal cancer is worthy of further exploration, beyond tumours with MSI or *POLE* mutation. Other genome-wide molecular phenotypes, such as mutational signatures,^28^ are also likely to have a role in cancer management in the future.

## References

[1] Guinney J, Dienstmann R, Wang X (2015). The consensus molecular subtypes of colorectal cancer. Nat Med.

[2] Boland CR, Goel A (2010). Microsatellite instability in colorectal cancer. Gastroenterology.

[3] Popat S, Hubner R, Houlston RS (2005). Systematic review of microsatellite instability and colorectal cancer prognosis. J Clin Oncol.

[4] Domingo E, Freeman-Mills L, Rayner E (2016). Somatic *POLE* proofreading domain mutation, immune response, and prognosis in colorectal cancer: a retrospective, pooled biomarker study. Lancet Gastroenterol Hepatol.

[5] Venderbosch S, Nagtegaal ID, Maughan TS (2014). Mismatch repair status and *BRAF* mutation status in metastatic colorectal cancer patients: a pooled analysis of the CAIRO, CAIRO2, COIN, and FOCUS studies. Clin Cancer Res.

[6] Dienstmann R, Mason MJ, Sinicrope FA (2017). Prediction of overall survival in stage II and III colon cancer beyond TNM system: a retrospective, pooled biomarker study. Ann Oncol.

[7] Phipps AI, Limburg PJ, Baron JA (2015). Association between molecular subtypes of colorectal cancer and patient survival. Gastroenterology.

[8] Schell MJ, Yang M, Teer JK (2016). A multigene mutation classification of 468 colorectal cancers reveals a prognostic role for APC. Nat Commun.

[9] Seppala TT, Bohm JP, Friman M (2015). Combination of microsatellite instability and *BRAF* mutation status for subtyping colorectal cancer. Br J Cancer.

[10] Sinicrope FA, Shi Q, Smyrk TC (2015). Molecular markers identify subtypes of stage III colon cancer associated with patient outcomes. Gastroenterology.

[11] Taieb J, Zaanan A, Le Malicot K (2016). Prognostic effect of *BRAF* and *KRAS* mutations in patients with stage III colon cancer treated with leucovorin, fluorouracil, and oxaliplatin with or without cetuximab: a post hoc analysis of the PETACC-8 trial. JAMA Oncol.

[12] Cancer Genome Atlas (2012). Comprehensive molecular characterization of human colon and rectal cancer. Nature.

[13] Kerr RS, Love S, Segelov E (2016). Adjuvant capecitabine plus bevacizumab versus capecitabine alone in patients with colorectal cancer (QUASAR 2): an open-label, randomised phase 3 trial. Lancet Oncol.

[14] Allegra CJ, Yothers G, O'Connell MJ (2013). Bevacizumab in stage II-III colon cancer: 5-year update of the National Surgical Adjuvant Breast and Bowel Project C-08 trial. J Clin Oncol.

[15] De Gramont A, Van Cutsem E, Schmoll HJ (2012). Bevacizumab plus oxaliplatin-based chemotherapy as adjuvant treatment for colon cancer (AVANT): a phase 3 randomised controlled trial. Lancet Oncol.

[16] McShane LM, Altman DG, Sauerbrei W (2005). Reporting recommendations for tumor marker prognostic studies (REMARK). J Natl Cancer Inst.

[17] Domingo E, Ramamoorthy R, Oukrif D (2013). Use of multivariate analysis to suggest a new molecular classification of colorectal cancer. J Pathol.

[18] Gupta S, Artomov M, Goggins W, Daly M, Tsao H (2015). Gender disparity and mutation burden in metastatic melanoma. J Natl Cancer Inst.

[19] Strickland KC, Howitt BE, Shukla SA (2016). Association and prognostic significance of *BRCA1/2*-mutation status with neoantigen load, number of tumor-infiltrating lymphocytes and expression of PD-1/PD-L1 in high grade serous ovarian cancer. Oncotarget.

[20] Birkbak NJ, Kochupurakkal B, Izarzugaza JM (2013). Tumor mutation burden forecasts outcome in ovarian cancer with *BRCA1* or *BRCA2* mutations. PLoS One.

[21] Snyder A, Makarov V, Merghoub T (2014). Genetic basis for clinical response to CTLA-4 blockade in melanoma. N Engl J Med.

[22] Rizvi NA, Hellmann MD, Snyder A (2015). Cancer immunology. Mutational landscape determines sensitivity to PD-1 blockade in non-small cell lung cancer. Science.

[23] Chalmers ZR, Connelly CF, Fabrizio D (2017). Analysis of 100,000 human cancer genomes reveals the landscape of tumor mutational burden. Genome Med.

[24] Chen JH, Zhang P, Chen WD (2015). ATM-mediated PTEN phosphorylation promotes PTEN nuclear translocation and autophagy in response to DNA-damaging agents in cancer cells. Autophagy.

[25] Sforza V, Martinelli E, Ciardiello F (2016). Mechanisms of resistance to anti-epidermal growth factor receptor inhibitors in metastatic colorectal cancer. World J Gastroenterol.

[26] Kawakami H, Zaanan A, Sinicrope FA (2015). Microsatellite instability testing and its role in the management of colorectal cancer. Curr Treat Options Oncol.

[27] Le DT, Durham JN, Smith KN (2017). Mismatch repair deficiency predicts response of solid tumors to PD-1 blockade. Science.

[28] Alexandrov LB, Nik-Zainal S, Wedge DC (2013). Signatures of mutational processes in human cancer. Nature.

