# Supplementary material for: Mutation burden and other molecular markers of prognosis in colorectal cancer treated with curative intent: results from the QUASAR 2 clinical trial and an Australian community-based series
Source: Lancet Gastroenterol Hepatol. 2018 Jul 2;3(9):635–43. doi: 10.1016/S2468-1253(18)30117-1 (PMC6088509; doi:10.1016/S2468-1253(18)30117-1)
Supplement: Supplementary appendix [file mmc1.pdf]

THE LANCET
Gastroenterology & Hepatology

Supplementary appendix

This appendix formed part of the original submission and has been peer reviewed. We post it as supplied by the authors.

Supplement to: Domingo E, Camps C, Kaisaki PJ, et al. Mutation burden and other molecular markers of prognosis in colorectal cancer treated with curative intent: results from the QUASAR 2 clinical trial and an Australian community-based series. *Lancet Gastroenterol Hepatol* 2018; published online July 2. http://dx.doi.org/10.1016/S2468-1253(18)30117-1.

SUPPLEMENTARY DATA

Supplementary Materials and Methods

Molecular screening and analysis in QUASAR2

FFPE tumour blocks, bloods and DNAs were stored at room temperature, -80°C and -20°C respectively. As previously described[1], MSI was analysed using all 5 Bethesda microsatellite markers (BAT15, BAT26, D2S123, D5S356 and D17S250)[2] and BAT40 (Supp Table 5). Tumours were classified as MSI+ if they had 40% or more unstable markers, and otherwise as MSI-.

Mutations in codons 12 and 13 of *KRAS* and *BRAF* V600E were analysed by direct DNA sequencing (Supp Table 5) and visualised with Mutation Surveyor software (Softgenetics). Somatic *POLE* mutations were assessed by allele-specific PCR (Taqman, ThermoFisher, UK) at three hotspots (p.Pro286Arg, p.Val411Leu and p.Ser459Phe)[3]. Additionally, two patients negative for these mutations but showing evidence of hypermutation (see Panel Sequencing section below) were further screened for the whole *POLE* gene by targeted sequencing. Both normal and tumour of these two patients, but no control samples, showed a p.Leu424Val mutation (data not shown) which is a known high risk allele predisposing to CRC[4]. Validation of this variant was performed by allele-specific PCR.

CIN analysis was performed as previously reported[5] with minor changes. Sections were enzymatically digested (Sigma protease, type XXIV, Sigma Chemical, St. Louis) for the preparation of monolayers, using a modification of Hedley's method[6]. The monolayers were stained with the Feulgen Basic Fuschin method. DNA content was measured using a work station (PWS, Room4, Sussex, UK) composed of an Axioplan II brightfield microscope (Zeiss, Germany) equipped with a scanning stage and controller (Prior scientific, UK), a Piezo focusing unit (PI, Germany) and a 10 bit CCD camera (Zeiss, Germany), a PCDIG frame card (Correco Imaging, USA) and a 546 nm green filter. 1500 nuclei were automatically captured and measured in each case. To exclude possible artefacts and non-representative nuclei like doublets, necrotic or cut cells, all images of nuclei were certified by trained personnel. DNA histograms (frequency distribution of nuclei, scaled from internal reference cells) were classified in a blinded manner. A sample was considered to be diploid if only one G_0/G_1 peak was present and the number of cells in the G_2 peak did not exceed 10%. If the G_2 peak was more than 10%, or a peak was present in the 8c position, the sample was classified as tetraploid. The sample was considered aneuploid when a peak appeared in an area outside 2c, 4c and 8c, or when the number of euploid nuclei exceeding 5c was over 1%. This cut-off was estimated on the basis of 50 random non-tumour specimens from the same patient group. Approximately 30% of the diploid histograms had nuclei exceeding 5c, but none of them over 1%. Lymphocytes, plasma cells and fibroblasts from each specimen were included as reference cells. Results were grouped into diploid (CIN-) or non-diploid (CIN+) categories for subsequent analyses.

A customized gene panel of 82 genes – including *KRAS, BRAF* and other major CRC drivers – was created using the Ion AmpliSeq Designer (*Thermo Fisher*). The inclusion criteria for the genes were decided by a consensus expert group based on major cancer driver genes in solid tumours and genes potentially relevant for targeted therapy (Supp. Table

3a). 3,126 primer pairs generating amplicons of ~100bp were designed to cover all coding areas of the genes concerned. All designs covered >95% of the target region. All probe sets were evaluated *in silico* and the TargetSeq probe set was subsequently redesigned, allowing for up to 8 close matches between the probe and build hg19 of the human genome (as opposed to the default setting of 5).

Libraries were generated according to the manufacturer's protocol. Briefly, multiplex PCR was performed with the Ion Ampliseq library kit 2.0 using 10 ng DNA for each of two primer pools, which were combined after target amplification. IonXpress-barcoded adapters were attached to the amplicons by ligation. The libraries were purified using Agencourt AMPure XP magnetic beads (Beckman Coulter Ltd, High Wycombe, UK) and amplified using adapter primers. The amplified libraries were purified again and quantified on an Agilent 2100 Bioanalyzer using the High Sensitivity DNA kit (Agilent Technologies, Stockport, UK). Library templates for sequencing were prepared by emulsion PCR on the One Touch 2 instrument and loaded onto 318 semiconductor chips and the Ion Torrent PGM™ sequencer[7]. One tumour-normal pair was run per chip, with a ratio of 10:1, in order to obtain an average coverage of 1000X for tumour and 100X for normal samples. 99% of amplicons within both the tumour and normal data met these criteria. Overall, 597 tumour-normal pairs were sequenced.

Mutation quality control

Somatic variants were called by Ion Reporter Software v4.0 (*Thermo Fisher*) using the default Tumour-Normal workflow. Filtering out of low quality variants was performed in three stages. Firstly, variants were removed if marked as low confidence by Ion Reporter (allele frequencies <10% in both tumour and normal or low coverage in the normal) or were CNVs, off-target variants, or indels with more than one alternative variant.

Secondly, in order to remove artefacts, including deamination that results in C:G>T:A substitutions[8], a predictive regression model with true or false positive mutation as the outcome was built, based on a subset of variants classified with high confidence as true and false positives. True positives were identified from main mutation hotspots in 10 CRC driver genes (Supp. Table 6) and false positives were defined heuristically as those non-synonymous variants found more frequently than *BRAF* V600E (Supp. Table 7), the most common single nucleotide change in CRC[9]. Predictive variables in the model included number of reads supporting the variant, allelic frequency in the tumour, allelic frequency in the normal and a P-value for quality provided by Ion Reporter (Supp. Table 8). All four variables were independently associated with the outcome. Accordingly, coefficients and constant were applied in the standard logistic regression model to all reported variants with the following formula:

$$\ln(P/1\text{-}P) = \beta_0 + \beta_1 X_1 + \beta_2 X_2 + \ldots + \beta_k X_K$$

where P is the probability that a variant is a true positive and *K* in this instance=1-4. A ROC curve was then plotted (Supp. Figure 2) where P≥0.39 gave the highest number of correctly classified variants (95.4%), corresponding to high predicted sensitivity (96.7%) and specificity (94.5%). Variants with P<0.39 were therefore excluded from analysis.

Thirdly, in order to take a conservative approach, we excluded cancers with very high levels of deamination and hence high levels of false-positive mutations despite the above variant filtering. In a plot of total mutation burden against proportion of C:G>T:A changes in both our data and CRC TGCA data, we applied a heuristic cut-off (Supp. Figure 4) and removed 86 cancers (14.4%).

We then performed three different analyses to check the quality of our mutations. Firstly, we found mutation spectra resembling the three main signatures reported for CRC linked to aging, MSI and *POLE* (signatures 1, 6 and 10 respectively) in the overall CRC sample set[10] (Supp. Figure 5). Secondly, the levels of hypermutation for SNVs and indels according to MSI and *POLE* status were in agreement with the literature[9,11] (Supp. Table 11 and Supp. Figure 6). Finally, our mutations in *KRAS* and *BRAF* from Sanger sequencing and Ion Torrent showed 97% concordant results. In summary, all three tests suggested that a high quality set of mutations remained after filtering using our sequencing and subsequent downstream pipeline analysis.

Mutation burden

The mutation burden of each cancer was measured as the total number of somatic non-synonymous variants and coding indels affecting any gene in the panel, after applying quality control. This measure of burden was very strongly correlated with the number of all mutations, including synonymous and extra-exonic changes (r^2=0.98) (Supp Figure 11). Both measures gave equivalent results in the analyses (data not shown). Quartiles were calculated such that ties (burden values lying on a quartile boundary) were assigned to the same quartile, specifically to the quartile with the larger number of cancers with that burden.

Driver mutation assignment

In order to classify variants as putative driver mutations, we first identified genes in which recurrent pathogenic mutations have recently been reported in a pan-cancer analysis[12], or are known to be *bona fide* tumour suppressors from the wider literature. From the resulting 25 genes, mutations were selected as drivers as follows: 1- nonsense, frameshift or splice site mutations in tumour suppressor genes; 2- hotspot missense reported as pathogenic according to Chang et al[12], including across homologues (e.g. Ras genes); 3- mutations predisposing to colorectal cancer according to ClinVar (rated with at least 2 stars and manually curated); 4- additional *TP53* mutations showing less than 50% expression in yeast compared to wildtype in all assays according to the IARC database[13] (Supp. Table 9). Exploratory analyses with or without these few mutations gave extremely similar results (data not shown). Any driver mutation with a variant allele frequency (VAF) below 0.1 with a minimum read depth of 100X, from a cancer with a putative clonal mutation peak at VAF>0·25 was regarded as at low allele frequency and hence potentially sub-clonal. Such mutations were analysed for clonal diversity.

Association analysis

Methods for analysis of association (regression, clustering and Bayesian networks) have already been reported[14]. Briefly, pairwise associations for categorical and continuous variables were assessed using Fisher's exact test and Student's *t*-test, respectively. To control for multiple hypothesis testing, *P* values were corrected with the false discovery rate control proposed by Benjamini and Hochberg and the obtained *q* value was considered significant if <0.05. Multivariate analysis was performed using both reverse and forward stepwise logistic regression (or linear regression for age and cancer differentiation) and $P < 0.05$ considered significant.

Additionally, cluster analyses were performed, with the aim of finding robust associations and pathways using more than one method. Unsupervised hierarchical agglomerative clustering analysis was performed with Ward's method, in which the distance between two clusters is the increase in variance for the cluster being merged, and a heat map was created. To build a model of the relationships between variables to identify which variables depend on which others and which are conditionally independent, a static Bayesian network analysis was done. This provides heuristic search strategies, consisting of a proposer for a new network, a cycle checker which investigates proposed networks for cycles, an evaluator which calculates the scores of proposed networks, and a decider to accept or not a proposed network. All analyses were performed with STATA, R or Banjo.

Molecular screening in Australian cohort

Tissue and DNA were stored at -80°C and -20°C respectively. Targeted sequencing was performed with custom amplicon panels against 113 genes designed for use with the HaloPlex™ Target Enrichment System (Supp. Table 3b). Probes were designed using maximum stringency optimised criteria and only genes with >90% probe coverage were included in the final design. The final design was optimised for a read length of 100 bp. A total of 33,942 amplicons were sequenced, with total target coverage of 99·2% across the gene panel. Sample libraries were prepared using the automated Bravo liquid handling platforms optimised for HaloPlex™ Target enrichment in a 96 well format. This system was optimised for successful digestion, library indexing, and purification from a total input 225ng of DNA. The final library was sequenced on the NextSeq 500 (Illumina, USA) using the NextSeq500/550 V2 High Output Kit-150 cycles (Illumina, USA, FC-404-2002) as per manufacturer's instructions. Each sample was sequenced at ~2 million reads per sample. Both tumour and normal samples were sequenced to an average coverage of greater than 1000 reads, in all cases >100-fold coverage of >95% of targeted regions was achieved. Mutations were called using a modified GATK "Best Practice" pipeline, removing the "removal of duplication" step. Somatic mutations were identified by subtracting matched normal variants from those identified in the tumour. Mutation spectra (Supp. Figure 9) and levels of hypermutation according to MSI and *POLE* status (Supp. Figure 10) were in agreement with the literature[4,9,10].

MSI was analysed with tumour and matched normal DNA that was polymerase chain reaction (PCR)-amplified for the Bethesda panel of microsatellite markers (BAT25, BAT26, D2S123, D5S346, D17S250) using fluorescently-labelled

primers[1]. Reaction products were analysed on a 3130xl Genetic Analyzer (Applied Biosystems). MSI+ was diagnosed if instability was evident at two or more markers.

All patients of the cohort were characterised for *KRAS* (codons 12, 13), *TP53* (exons 4-9) and *BRAF* codon 600 mutations based on Sanger sequencing at the Ludwig Institute Parkville, Melbourne. Tumours were microdissected from the originating FFPE blocks. Hematoxylin and eosin–stained sections were reviewed by an anatomical pathologist and macrodissected in areas comprising greater than 60% neoplastic cells. Genomic DNA was extracted using the AllPrep DNA/RNA Mini Kit (Qiagen) and amplified using PCR (Supp. Table 5). Direct DNA sequencing was conducted using BigDye Terminator v3.1 Ready Reaction Mix (Applied Biosystems). Reaction products were run on 3730xl DNA Analyzers (Applied Biosystems). Mutations detected were confirmed as somatic by bidirectional resequencing of new PCR products from tumour and matched normal DNA or cell line DNA. Primer details are available from authors. Samples with indeterminate sequencing traces on first analysis were subjected to repeat PCR and sequencing.

Survival analyses

Time-to-event analysis was performed for relapse-free survival (RFS) right-truncated at 5 years, as biologically this is the most relevant endpoint. RFS was defined as time from surgery to the first confirmed relapse, with censoring done when a patient died from causes unrelated to cancer or was alive without recurrence at last contact. Kaplan–Meier survival curves were generated and univariable survival distributions compared. Cox proportional hazards models were used to estimate survival distributions and hazard ratios and multivariate analyses included T stage, N stage, treatment arm and MSI where relevant. All survival analyses were two-sided and considered significant if $P < 0.05$. These were conducted with STATA. In all cases, we compared biomarker associations between the two arms of QUASAR2, and no significant differences were found (details not shown). Equivalent analyses were performed for the Australian cohort. Prognostic models were compared by nested likelihood ratio test and robustness was assessed by repeating the analysis randomly omitting 10% of cases each time. Note that in statistical analyses with the Australian cohort, *BRAF* mutations other than V600E in QUASAR2 were not taken as pathogenic as they had not been screened. Analyses with or without these few mutations gave extremely similar results (data not shown). All molecular assays were performed blinded to clinico-pathological and survival data. Samples with missing data in any given multivariable model were not included (Supp. Table 2 and Supp. Figure 1).

Supplementary Tables

Supplementary Table 1. Previous studies of molecular prognostic markers in CRC that comprised >400 patients[16-29].
Where multivariable analysis was performed, those results are reported, although use of co-variables varies among studies. Some studies may be sub-sets of others. Only nominally significant associations are reported: *=P<0.05, **=P<0.01, ***=P<0.001 (all uncorrected values). MSI does not include "MSI-low" but may be variably defined. MMR=presence (P) or deficiency (D) of mismatch repair proteins, principally MLH1. [$]No P value reported; ^Compared with 3 other subtypes with similar trends ; [#]HR>1 denotes longer survival in that study.

Ref.	Study	N	Patients	Markers	Results (single marker:HR)	Results (subtypes:HR)	Analysis	Comments
Rui 2015	Meta-analysis	3771	Population or hospital CRC	KRAS	KRAS:0.78***#		OS	
Sinicrope 2015	NCCTG N0147	2720	Stage III colon cancer	KRAS, BRAF, MMR		KRAS/MMRP: 1.48***, BRAF/MMRP:1.43**	DFS, subtypes	Additional validation set (colon cancer, N=783)
Phipps 2015	Seattle registry	2050	Population CRC	MSI, CIMP, KRAS, BRAF		MSI-/CIMP-/KRAS-/BRAF- vs MSI-/CIMP+/KRAS-/BRAF+: HR2.20*$	CRC-specific survival, multivariable	Other subtypes reported
Taieb 2016	PETACC-8	1791	Stage III colon cancer	MMR, KRAS, BRAF	MSI:1.10, KRAS:1.55*, BRAF:1.22	MMRP/KRAS:1.64***, MMRP/BRAF:1.74**, MMRD/BRAF:0.23*	DFS, multivariable analysis	
Roth 2012	PETACC-3	1404	Stage II/III colon cancer	MSI, KRAS, BRAF	MSI: 0.54*** KRAS:1.05BRAF:1.17		RFS, multivariable	
Andre 2015	MOSAIC	1008	Stage II/III colon cancer	MMR, BRAF	MMRD:2.02* BRAF not reported		OS, multivariable bootstrap analysis	
Mouradov 2013	VICTOR	822	Stage II/III CRC	MSI, CIN, KRAS, BRAF, NRAS, PIK3CA, TP53, FBXW7	MSI:0.49*, CIN:1.63**, genes not significant		RFS, multivariable	Additional validation set (population CRC stage II/III, N=375)
Kadowaki 2015	Saitama Cancer Centre	813	Hospital stage I/II/III CRC	MSI, KRAS, BRAF	MSI:0.64, KRAS:1.35*, BRAF:2.20*		DFS	

Seppala 2015	Central Hospital Central Finland	762	Population CRC	MSI, BRAF	MSI:0.49**, BRAF:1.43**	MSI-/BRAF: 1.88*, MSI+/BRAF: 0.42*	DFS	
Jorissen 2015	Australian Hospitals	746	Incident CRC	APC		MSI-/APCwt/proximal:~2*^	RFS, multivariable	
Merok 2013	Aker	613	Hospital all stages CRC	MSI	MSI: 1.60*#		RFS, multivariable	
Dienstmann 2017	Aker	609	Hospital Stage II/III colon cancer			KRAS/MSI-: 1.43*, BRAF/MSI-:2.06**	OS, univariable	
Dienstmann 2017	Colorectal Cancer Subtyping Consortium	609	4 datasets Stage II/III colon cancer			KRAS/MSI-: 1.32*, BRAF/MSI-:1.75*	OS, univariable	
Schell 2016	Moffitt Cancer Centre	468	Population CRC	MSI, 1321 cancer genes		MSI-/APC:0.65*, MSI-/KRAS:1.62**; MSI-/BRAF:2.63**, MSI-/TP53:1.41	OS, multivariable analysis in subtypes	
Deng 2015	Sun Yat-sen University	433	Hospital stage II/III CRC	KRAS	KRAS:1.57*		DFS, all had FOLFOX	
Manceau 2015	Cartes d'Identité des Tumeurs	433	Hospital stage I/II/III colon cancer	MSI, PIK3CA		MSI-/PIK3CA:0.12**	RFS	Additional validation set (N=393)

Supplementary Table 2. Distribution of clinico-pathological variables in all cohorts.
Numbers of patients (%) are shown in each cell. Q2 NGS=QUASAR2 cancers analysed using Ion Torrent gene panel; Q2 extended=QUASAR2 cancers analysed by Sanger sequencing. Australia NGS=Australian cancers analysed using targeted NGS; Australia extended=Australian cancers analysed by Sanger sequencing. All Australia samples also had MSI assessed. Proximal location=caecum, ascending colon, hepatic flexure and transverse colon; distal location=splenic flexure, descending colon, sigmoid colon and rectum. For the Australian samples, data for radiotherapy were only available for those patients whose cancers that had been analysed using NGS.

		Q2 NGS	Q2 Extended	Australia NGS	Australia Extended	Total
**T**	**T1-3**	309 (65)	378 (65)	228 (77)	299 (60)	1214 (69)
	**T4**	202 (35)	206 (35)	68 (23)	62 (40)	538 (31)
**N**	**N0**	166 (32)	232 (40)	110 (37)	135 (37)	643 (37)
	**N1-2**	345 (68)	352 (60)	186 (63)	226 (63)	1109 (63)
**Gender**	**Male**	265 (52)	361 (62)	166 (56)	197 (55)	989 (56)
	**Female**	246 (48)	223 (38)	130 (44)	164 (45)	763 (44)
**Location**	**Right**	197 (39)	224 (38)	114 (39)	149 (41)	684 (39)
	**Left**	282 (55)	296 (51)	182 (61)	211 (58)	971 (55)
	**Unknown**	32 (6)	64 (11)	0	1 (1)	96 (5)
**Differentiation**	**Well/Moderate**	405 (79)	458 (78)	242 (82)	272 (75)	1377 (79)
	**Poor**	79 (15)	88 (15)	50 (17)	77 (21)	294 (17)
	**Unknown**	27 (5)	38 (7)	4 (1)	12 (3)	81 (4)
**Trial arm**	**Cap**	234 (46)	299 (51)	-	-	533 (49)
	**Cap+Bev**	277 (54)	285 (49)	-	-	562 (51)
**Chemotherapy**	**No**	-	-	131 (44)	147 (41)	278 (42)
	**Yes**	-	-	165 (56)	214 (59)	379 (58)
**Radiotherapy**	**No**	511 (100)	584 (100)	264 (89)	0	1359 (78)
	**Yes**	0	0	11 (4)	0	11 (1)
	**Unknown**	0	0	21 (7)	361 (100)	382 (22)
**Age (range)**	**Mean**	63.6 (27-85)	64.2 (21-83)	67.2 (29-94)	67.8 (30-92)	65.2 (21-94)

Supplementary Table 3. Genes in targeted sequencing panels.

(a) QUASAR2

ABL1	DDR2	IDH2	PTEN	WT1
AKT1	EGFR	JAK2	PTPN11	XRCC2
ALK	ERBB2	JAK3	RAD51	
APC	ERBB4	KDR	RB1	
AR	ERCC1	KIT	RET	
ATM	EZH2	KRAS	RRM1	
ATRX	FANCA	MAP2K1	RUNX1	
AURKA	FANCC	MAP2K2	SMAD4	
AURKB	FBXW7	MAP3K1	SMARCB1	
AURKC	FGFR1	MDM2	SMO	
BRAF	FGFR2	MET	SRC	
BRCA1	FGFR3	MLH1	STK11	
BRCA2	FLT3	MTOR	TET2	
C11orf30	GNAS	N4BP2L2	TLR4	
CCND1	HDAC1	NF1	TNKS1	
CDH1	HDAC2	NOTCH1	TNKS2	
CDKN2A	HDAC3	NPM1	TP53	
CSF1R	HNF1A	NRAS	TTLL5	
CTNNB1	HRAS	PDGFRA	UGT1A1	
CYP2D6	IDH1	PIK3CA	VHL	

(b) Australian cohort

ACVR2A	CDC42EP1	GNRH2	MESP1	PTEN	TGIF1
ADCK5	CHD6	GPX1	MICALCL	PTPLA	TIAM1
ADM2	CNOT2	GRIN3B	KMT2D	RALY	TMEM184A
ADRA2B	CREBBP	GSPT1	MUC6	RAVER1	TMPRSS13
AP3S1	CTNNB1	HDGFRP2	MYADML2	RBM5	TP53
APC	CTSA	HNRNPL	NEUROG3	RIN3	TRRAP
ASH1L	DBR1	HRCT1	NRAS	RNF43	TTLL11
ATM	DIAPH1	HTT	OVGP1	RNPC3	TYRO3
ATN1	DRD4	IRF5	PABPC1	RPL14	WRAP53
ATXN1	ECHS1	KANK3	PAK2	SAFB2	ZFHX3
AXIN2	ELF3	KDM6B	PCGF6	SLC5A10	ZFP36L2
B2M	EP300	KIAA0040	PHF2	SMAD4	ZNF787
BAIAP2L2	EP400	KMT2C	PHF21A	SOX9	ZNF880
BCL9L	ERBB3	KRAS	PIK3C2B	SPERT	
BZRAP1	FAM171B	KRTAP5-5	PIK3CA	SPHK1	
C21ORF58	FAM46A	LATS2	PLIN4	SPPL2B	
C6ORF223	FAT1	MAFA	PPM1E	SRCAP	
CACNA1B	FBXW7	MAL2	PRDM2	TBP	
CD58	FNBP4	MED31	PRIM2	TCF7L2	
CD7	GLTPD2	MEF2A	PRX	TDG	

Supplementary Table 4. Prognostic analyses performed and reported in this study.
The endpoint of all analyses was relapse-free survival (RFS). **GNAS* mutation was not available in the Australia cohort.

Prognostic Analysis	Cohort	Method	Reported
Selection of candidate biomarkers from targeted NGS	QUASAR2	Cox Proportional Hazards Model Univariable	Supp. Tables 15 & 16
Impact of *KRAS, BRAF, TP53*, *GNAS** and mutation burden	QUASAR2, Australia separately and pooled (with or without hypermutation)	Cox Proportional Hazards Model Univariable/Multivariable, Kaplan-Meier curves	Main text, Tables 1,2,3, Figure 1
Impact of *KRAS, BRAF, TP53*, and mutation burden according to disease stage	Pooled QUASAR2 and Australia (with or without hypermutation) in stage II and III separately	Cox Proportional Hazards Model Univariable and Multivariable	Supp. Table 18
Impact of *KRAS, BRAF, TP53*, and mutation burden according to location	Pooled QUASAR2 and Australia (with or without hypermutation) in proximal colon, distal colon and rectum separately	Cox Proportional Hazards Model Univariable/Multivariable	Supp. Table 19
Impact of combination of *KRAS, BRAF* and MSI	Pool of Extended QUASAR2 and Extended Australia without missing data	Cox Proportional Hazards Model Univariable/Multivariable, Kaplan-Meier curves	Main text, Table 4, Supp. Table 20, Figure 2
Impact of combination of *KRAS, BRAF* and *TP53* in MSI-	Pool of MSI- CRCs from QUASAR2 and Extended Australia without missing data	Cox Proportional Hazards Model Univariable/Multivariable, Kaplan-Meier curves	Main text, Supp. Table 21, Figure 3

Supplementary Table 5. Primers and conditions used for PCRs in QUASAR2 and Australian cohort.

Cohort	Amplicon	Forward Primer (5' to 3')	Reverse Primer (5' to 3')	Product Size (bp)	Annealing Temp.	Taq
QUASAR2	*KRAS* codons12/13	GTGTGACATGTTCTAATATAGTCA	GAATGGTCCTGCACCAGTAA	214	60	Qiagen Multiplex PCR kit
QUASAR2	*BRAF* codon 600	TGCTTGCTCTGATAGGAAAATG	CCACAAAATGGATCCAGACA	173	60	Qiagen Multiplex PCR kit
QUASAR2	BAT25	TCGCCTCCAAGAATGTAAGT	TCTGGATTTTAACTATGGCTC	~124	60	Qiagen Multiplex PCR kit
QUASAR2	BAT26	TGACTACTTTTGACTTCAGCC	TTCTTCAGTATATGTCAATGAAAACA	~154	60	Qiagen Multiplex PCR kit
QUASAR2	D2S123	AAACAGGATGCCTGCCTTTA	GGACTTTCCACCTATGGGAC	~211	60	Qiagen Multiplex PCR kit
QUASAR2	D5S346	ACTCACTCTAGTGATAAATCGGG	AGCAGATAAGACAGTATTACTAGTT	~125	58	Qiagen Multiplex PCR kit
QUASAR2	D17S250	GGAAGAATCAAATAGACAAT	GCTGGCCATATATATATTTAAACC	~152	58	Qiagen Multiplex PCR kit
QUASAR2	BAT40	CCTACACCACAACCCTGCTT	TGAGGTGGGAGGATAAATGG	~198	58	Qiagen Multiplex PCR kit
Australia	TP53_00300_1004	TGTAAAACGACGGCCAGTTCTGAGCAGCGCTCATGGT	CAGGAAACAGCTATGACCTCCTGAGGTGTAGACGCCAA	326	60	Life Technologies AmpliTaq Gold
Australia	TP53_00300_5006	TGTAAAACGACGGCCAGTGAGGGGCCAGACCTAAGAGC	CAGGAAACAGCTATGACCGCTTTATCTGTTCACTTGTGCCC	335	60	Life Technologies AmpliTaq Gold
Australia	TP53_00300_5005	TGTAAAACGACGGCCAGTTTTGCACATCTCATGGGGTT	CAGGAAACAGCTATGACCCTCAGATAGCGATGGTGAGC	338	60	Life Technologies AmpliTaq Gold
Australia	TP53_00200_5003	TGTAAAACGACGGCCAGTTGTGATGAGAGGTGGATGGGTA	CAGGAAACAGCTATGACCGCCTCCCCTGCTTGCCAC	302	60	Life Technologies AmpliTaq Gold
Australia	TP53_00100_5002	TGTAAAACGACGGCCAGTAGGAGCTGGTGTTGTTGGGC	CAGGAAACAGCTATGACCGGGTGGTTGGGAGTAGATGGA	350	60	Life Technologies AmpliTaq Gold
Australia	TP53_00100_1001	TGTAAAACGACGGCCAGTAAATGCCCCAATTGCAGGTAA	CAGGAAACAGCTATGACCGAATCTCCGCAAGAAAGGGG	324	60	Life Technologies AmpliTaq Gold
Australia	TP53_00000_1000	TGTAAAACGACGGCCAGTCCCTGGGTTTGGATGTTCTG	CAGGAAACAGCTATGACCTCTCCCCCTCCTCTGTTGCT	346	60	Life Technologies AmpliTaq Gold
Australia	KRAS_00100_4001	TGTAAAACGACGGCCAGTTGCACAGAGAGTGAACATCATGG	CAGGAAACAGCTATGACCTTTGAGAGCCTTTAGCCGCC	670	60	Life Technologies AmpliTaq Gold
Australia	KRAS_00000_4000	TGTAAAACGACGGCCAGTCCAGCACCACCACTACCGAT	CAGGAAACAGCTATGACCGTGGCCATTTGTCCGTCATC	550	60	Life Technologies AmpliTaq Gold

Australia	BRAF_00100_4001	TGTAAAACGACGGCCAGTTAGGAGTCCCGACTGCTGTG	CAGGAAACAGCTATGACCGGGACCTGTCATGGGGTAGG	706	60	Life Technologies AmpliTaq Gold
Australia	BRAF_00000_4000	TGTAAAACGACGGCCAGTGGGTACACAGAACATTTTGAACACA	CAGGAAACAGCTATGACCCACATTTCAAGCCCCAAAAA	745	60	Life Technologies AmpliTaq Gold
Australia	BAT26 F	TGACTACTTTTGACTTCAGCC	AACCATTCAACATTTTTAACCC	80-100	60	Life Technologies AmpliTaq Gold
Australia	D17S250-F	GGAAGAATCAAATAGACAAT	GCTGGCCATATATATATTTAAACC	151	55	Life Technologies AmpliTaq Gold
Australia	BAT25-F	TCGCCTCCAAGAATGTAAGT	TCTGCATTTTAACTATGGCTC	115-131	55	Life Technologies AmpliTaq Gold
Australia	D2S123-F	AAACAGGATGCCTGCCTTTA	GGACTTTCCACCTATGGGAC	197-227	55	Life Technologies AmpliTaq Gold
Australia	D5S346 F	ACTCACTCTAGTGATAAATCGGG	AGCAGATAAGACAGTATTACTAGTT	96-122	55	Life Technologies AmpliTaq Gold

Supplementary Table 6. Mutations considered true positives in QUASAR2 targeted sequencing
These were chosen as high-confidence somatic mutations for use in the regression model to clean false positive variants from the Ion Torrent sequencing. These are all very well described hotspots in CRC driver genes. See Supplementary Methods for further details.

#	Gene	Transcript		Exon	Chromosome	AA change
1	APC	NM_000038		6	c.C637T	p.R213X
2	APC	NM_000038		7	c.C646T	p.R216X
3	APC	NM_000038		7	c.C694T	p.R232X
4	APC	NM_000038		9	c.C847T	p.R283X
5	APC	NM_000038		16	c.C2413T	p.R805X
6	APC	NM_000038		16	c.C2626T	p.R876X
7	APC	NM_000038		16	c.C3340T	p.R1114X
8	APC	NM_000038		16	c.C4012T	p.Q1338X
9	APC	NM_000038		16	c.C4348T	p.R1450X
10	BRAF	NM_004333		15	c.T1799A	p.V600E
11	CTNNB1	NM_001904		3	c.C98G	p.S33C
12	CTNNB1	NM_001904		3	c.A121G	p.T41A
13	CTNNB1	NM_001904		3	c.T133C	p.S45P
14	CTNNB1	NM_001904		3	c.C134T	p.S45F
15	FBXW7	NM_033632		9	c.C1393T	p.R465C
16	FBXW7	NM_033632		9	c.G1394A	p.R465H
17	FBXW7	NM_033632		10	c.G1436A	p.R479Q
18	FBXW7	NM_033632		10	c.C1513T	p.R505C
19	KRAS	NM_033360		2	c.G35C	p.G12A
20	KRAS	NM_033360		2	c.G34T	p.G12C
21	KRAS	NM_033360		2	c.G35A	p.G12D
22	KRAS	NM_033360		2	c.G34C	p.G12R
23	KRAS	NM_004985		2	c.G34A	p.G12S
24	KRAS	NM_033360		2	c.G35T	p.G12V
25	KRAS	NM_033360		2	c.G38A	p.G13D
26	KRAS	NM_033360		3	c.A183C	p.Q61H
27	KRAS	NM_033360		3	c.A183T	p.Q61H
28	KRAS	NM_033360		3	c.C181A	p.Q61K
29	KRAS	NM_033360		4	c.G436C	p.A146P
30	KRAS	NM_004985		4	c.G436A	p.A146T
31	KRAS	NM_033360		4	c.C437T	p.A146V
32	NRAS	NM_002524		2	c.G34T	p.G12C
33	NRAS	NM_002524		2	c.G35A	p.G12D
34	NRAS	NM_002524		2	c.G38A	p.G13D
35	NRAS	NM_002524		3	c.C181A	p.Q61K
36	PIK3CA	NM_006218		10	c.G1624A	p.E542K
37	PIK3CA	NM_006218		10	c.G1633A	p.E545K
38	PIK3CA	NM_006218		10	c.C1636A	p.Q546K
39	PIK3CA	NM_006218		21	c.A3140G	p.H1047R
40	PTEN	NM_000314		5	c.G389A	p.R130Q
41	SMAD4	NM_005359		9	c.C1081T	p.R361C
42	SMAD4	NM_005359		9	c.G1082A	p.R361H
43	TP53	NM_001126114		5	c.G524A	p.R175H
44	TP53	NM_001126114		6	c.C586T	p.R196X
45	TP53	NM_001126114		6	c.C637T	p.R213X
46	TP53	NM_001126114		7	c.G733A	p.G245S
47	TP53	NM_001126114		7	c.G743A	p.R248Q
48	TP53	NM_001126114		7	c.C742T	p.R248W
49	TP53	NM_001126114		8	c.C817T	p.R273C
50	TP53	NM_001126114		8	c.G818A	p.R273H
51	TP53	NM_001126114		8	c.C844T	p.R282W
52	TP53	NM_001126114		8	c.C916T	p.R306X

Supplementary Table 7. Mutations considered false positives in QUASAR2 targeted sequencing
See Supplementary Methods for details.

Gene ID	Chromosome	Position	Ref	Alt	Counts
RUNX1	21	36164736	T	G	174
ABL1	9	133760827	G	A	152
ATM	11	108196877	G	A	139
ATM	11	108196878	G	A	128
ABL1	9	133760828	G	A	114
CCND1	11	69458003	G	A	113
ATM	11	108196876	G	A	97
ABL1	9	133760832	G	A	92
NOTCH1	9	139402490	C	T	90

Supplementary Table 8. Regression model used to filter in high quality mutations in QUASAR2
See Supplementary Methods for details.

$P = 1/(1 + EXP(-((0.0147 * FAO) + (19.5 * FreqT) + (127.1 * FreqN) - 8338500 * p\text{-}value) - 0.518)))$

Variable	Coefficient	95% CI	P-value
**FAO**	0.0147	0.0087-0.0206	<0.001
**FreqT**	19.5	15.4-23.6	<0.001
**FreqN**	127.1	78.2-177.2	<0.001
**P-value**	-8338500	-9390200-7286700	<0.001
**Constant**	-0.518	-0.938-0.0982	0.016

Supplementary Table 9. Total numbers of driver mutations in driver genes in QUASAR2 set.
*One *APC* frameshift mutation downstream of the first SAMP repeat at codon 1580 was not taken as pathogenic; #TP53 missense mutations classified as pathogenic based on yeast assays in IARC database; ^*NRAS* mutation homologue to a pathogenic *KRAS* mutation (A146T); %Mutations from ClinVar considered pathogenic.

Gene	Nonsense in TSG	Frameshift in TSG	Splice site in TSG	Missense (Chang et al)	Missense (other)	Total
APC	314	143*	12	0	0	469
TP53	56	24	20	208	28#	336
KRAS	0	0	0	206	0	206
PIK3CA	0	0	0	101	0	101
BRAF	0	0	0	79	0	79
FBXW7	39	3	1	27	0	70
SMAD4	8	5	0	21	0	34
ATM	11	3	3	3	3%	23
PTEN	8	8	2	3	0	21
NF1	12	6	0	0	0	18
CTNNB1	0	0	0	14	0	14
GNAS	0	0	0	8	0	8
NRAS	0	0	0	7	1^	8
MLH1	2	0	1	0	4%	7
ERBB2	0	0	0	6	0	6
AKT1	0	0	0	5	0	5
MTOR	0	0	0	5	0	5
MAP2K1	0	0	0	3	0	3
CDKN2A	1	0	0	0	0	1
HRAS	0	0	0	1	0	1
IDH1	0	0	0	1	0	1
Total	451	192	39	698	36	1,416

Supplementary Table 10. Associations from QUASAR2 gene panel between driver mutations, mutation burden, MSI & clinico-pathological variables.

		MSI			APC			TP53			KRAS			PIK3CA		
		MSS	MSI+	q	Wt	Mut	q	Wt	Mut	q	Wt	Mut	q	Wt	Mut	q
**CIN**	Neg	89	38	3E-14												
	Pos	314	8													
**APC**	Wt	134	43	4E-8												
	Mut	316	18													
**TP53**	Wt	147	50	8E-12	87	110	6E-4									
	Mut	303	11		90	224										
**KRAS**	Wt	257	51	6E-5	131	177	5E-5	116	192	0.94						
	Mut	193	10		46	157		81	122							
**PIK3CA**	Wt	365	50	1	145	270	0.98	152	263	0.28	263	152	0.03			
	Mut	85	11		32	64		45	51		45	51				
**BRAF**	Wt	403	29	8E-12	111	321	4E-20	158	274	0.15	230	202	3E-16	345	87	0.29
	Mut	47	32		66	13		39	40		78	1		70	9	
**FBXW7**	Wt	390	55	0.86	161	284	0.26	178	267	0.32	269	176	1.00	368	77	0.17
	Mut	60	6		16	50		19	47		39	27		47	19	
**SMAD4**	Wt	422	57	0.98	159	320	0.07	185	294	1.00	286	193	0.68	386	93	0.56
	Mut	28	4		18	14		12	20		22	10		29	3	
**ATM**	Wt	431	58	0.96	169	320	0.98	182	307	0.04	296	193	0.95	400	89	0.43
	Mut	19	3		8	14		15	7		12	10		15	7	
**NF1**	Wt	435	58	0.79	171	322	1.00	186	307	0.21	298	195	0.98	401	92	0.97
	Mut	15	3		6	12		11	7		10	8		14	4	
**PTEN**	Wt	436	57	0.57	169	324	0.78	187	306	0.43	295	198	0.67	401	92	0.97
	Mut	14	4		8	10		10	8		13	5		14	4	
**CTNNB1**	Wt	444	54	3E-3	167	331	0.02	187	311	0.04	302	196	0.71	404	94	1.00
	Mut	6	7		10	3		10	3		6	7		11	2	
**GNAS**	Wt	445	58	0.23	174	329	1.00	190	313	0.04	305	198	0.60	410	93	0.46
	Mut	5	3		3	5		7	1		3	5		5	3	
**NRAS**	Wt	442	61	0.93	175	328	0.96	192	311	0.60	300	203	0.12	408	95	1.00
	Mut	8	0		2	6		5	3		8	0		7	1	
**Burden**	Q1	154	3	1E-18	75	82	8E-9	59	98	0.09	110	47	0.001	142	15	0.003
	Q2	155	6		39	122		50	111		86	75		135	26	
	Q3	69	9		17	61		31	47		32	46		52	26	
	Q4	72	43		46	69		57	58		80	35		86	29	
**T**	T1-3	277	32	0.51	96	213	0.16	110	199	0.31	194	115	0.45	249	60	0.96
	T4	173	29		81	121		87	115		114	88		166	36	
**Stage**	II	140	26	0.28	60	106	0.93	77	89	0.08	95	71	0.67	139	27	0.67
	III	310	35		117	228		120	225		213	132		276	69	
**Gender**	Male	237	28	0.67	84	181	0.43	105	160	0.94	166	99	0.60	213	52	0.94
	Female	213	33		93	153		92	154		142	104		202	44	
**Site**	Prox.	148	49	2E-10	96	101	2E-6	94	103	0.01	116	81	0.96	154	43	0.49
	Distal	271	11		212	212		191	191		110	110		48	48	
**Diffn**	Well	42	7	2E-8	19	30	0.08	22	27	0.09	26	23	0.60	41	8	0.37
	Mod.	332	24		111	245		123	233		214	142		280	76	
	Poor	51	28		38	41		40	39		53	26		70	9	
**Age**	Mean	63.5	64.4	0.83	63.6	63.5	0.86	63.6	63.5	1.00	63.2	64.2	0.56	63.6	63.4	1.00

		BRAF			FBXW7			SMAD4			ATM			NF1		
		Wt	Mut	q	Wt	Mut	q	Wt	Mut	q	Wt	Mut	q	Wt	Mut	q
**CIN**	Neg															
	Pos															
**APC**	Wt															
	Mut															
**TP53**	Wt															
	Mut															
**KRAS**	Wt															
	Mut															
**PIK3CA**	Wt															
	Mut															
**BRAF**	Wt															
	Mut															
**FBXW7**	Wt	376	69	1.00												
	Mut	56	10													
**SMAD4**	Wt	412	67	0.01	416	63	0.98									
	Mut	20	12		29	3										
**ATM**	Wt	415	74	0.68	427	62	0.83	458	31	1.00						
	Mut	17	5		18	4		21	1							
**NF1**	Wt	416	77	1.00	430	63	0.96	461	32	0.93	472	21	0.86			
	Mut	16	2		15	3		18	0		17	1				
**PTEN**	Wt	419	74	0.46	430	63	0.96	462	31	1.00	473	20	0.46	479	14	0.02
	Mut	13	5		15	3		17	1		16	2		14	4	
**CTNNB1**	Wt	419	79	0.56	432	66	0.71	466	32	1.00	477	21	0.77	480	18	1.00
	Mut	13	0		13	0		13	0		12	1		13	0	
**GNAS**	Wt	427	76	0.35	439	64	0.60	472	31	0.74	481	22	1.00	486	17	0.57
	Mut	5	3		6	2		7	1		8	0		7	1	
**NRAS**	Wt	425	78	1.00	440	63	0.26	471	32	1.00	481	22	1.00	488	15	0.02
	Mut	7	1		5	3		8	0		8	0		5	3	
**Burden**	Q1	142	15	1E-5	154	3	2E-6	151	6	0.65	156	1	0.047	157	0	0.007
	Q2	143	18		136	25		148	13		154	7		157	4	
	Q3	70	8		65	13		74	4		73	5		72	6	
	Q4	77	38		90	25		106	9		106	9		107	8	
**T**	T1-3	267	42	0.45	266	43	0.75	296	13	0.12	295	14	0.99	297	12	0.93
	T4	165	37		179	23		183	19		194	8		196	6	
**Stage**	II	142	24	0.96	146	20	0.97	155	11	1.00	161	5	0.68	160	6	1.00
	III	290	55		299	46		324	21		328	17		333	12	
**Gender**	Male	239	26	4E-4	228	37	0.83	250	15	0.91	252	13	0.84	254	11	0.80
	Female	193	53		217	29		229	17		237	9		239	7	
**Site**	Prox.	138	59	6E-11	173	24	0.95	182	15	0.67	186	11	0.43	187	10	0.37
	Distal	17	17		39	39		15	15		8	8		6	6	
**Diffn**	Well	40	9	0.02	47	2	0.04	42	7	0.27	47	2	0.95	45	4	0.23
	Mod.	312	44		313	43		336	20		343	13		348	8	
	Poor	57	22		61	18		75	4		75	4		76	3	
**Age**	Mean	63.2	65.5	0.14	63.4	64.7	0.64	63.6	63.5	1.00	63.6	63.5	0.99	63.6	61.4	0.77

		PTEN			CTNNB1			GNAS			NRAS			Mutn. burden				
		Wt	Mut	Q	Wt	Mut	q	Wt	Mut	q	Wt	Mut	q	Q1	Q2	Q3	Q4	q
**CIN**	Neg																	
	Pos																	
**APC**	Wt																	
	Mut																	
**TP53**	Wt																	
	Mut																	
**KRAS**	Wt																	
	Mut																	
**PIK3CA**	Wt																	
	Mut																	
**BRAF**	Wt																	
	Mut																	
**FBXW7**	Wt																	
	Mut																	
**SMAD4**	Wt																	
	Mut																	
**ATM**	Wt																	
	Mut																	
**NF1**	Wt																	
	Mut																	
**PTEN**	Wt																	
	Mut																	
**CTNNB1**	Wt	481	17	0.71														
	Mut	12	1															
**GNAS**	Wt	485	18	1.00	491	12	0.47											
	Mut	8	0		7	1												
**NRAS**	Wt	485	18	1.00	490	13	1.00	495	8	1.00								
	Mut	8	0		8	0		8	0									
**Burden**	Q1	155	2	0.019	154	3	0.49	157	0	0.21	155	2	0.98					
	Q2	159	2		159	2		159	2		158	3						
	Q3	71	7		76	2		76	2		76	2						
	Q4	108	7		109	6		111	4		114	1						
**T**	T1-3	299	10	0.98	302	7	0.97	307	2	0.24	302	7	0.43	98	102	42	67	0.77
	T4	194	8		196	6		196	6		201	1		59	59	36	48	
**Stage**	II	156	10	0.17	162	4	1.00	163	3	0.96	165	1	0.78	56	47	25	38	0.93
	III	337	8		336	9		340	5		338	7		101	114	53	77	
**Gender**	Male	257	8	0.93	256	9	0.60	265	0	0.02	263	2	0.43	80	90	39	56	0.91
	Female	236	10		242	4		238	8		240	6		77	71	39	59	
**Site**	Prox.	184	13	0.03	189	8	0.10	192	5	0.29	195	2	0.80	41	53	35	68	2E-7
	Distal	4	4		2	2		1	1		6	6		106	102	37	37	
**Diffn**	Well	48	1	1.00	49	0	0.07	48	1	0.93	48	1	0.80	17	10	8	14	0.069
	Mod.	343	13		350	6		352	4		349	7		119	118	53	66	
	Poor	76	3		73	6		78	1		79	0		14	28	11	26	
**Age**	Mean	63.8	58	0.04	63.7	59.3	0.35	63.6	64.9	0.96	63.6	62.1	0.95	63.6		63.5		0.77

Supplementary Table 11. Mutation (SNV and indel) burden in QUASAR2 by MSI and POLE status.

		SNVs			Indels		
Instability	N	Mean	SE	P	Mean	SE	P
MSI-/POLEwt	443	7.83	0.39	Ref	0.53	0.04	Ref
MSI+	61	19.43	1.38	1.07×10^{-21}	1.10	0.14	2.87×10^{-7}
POLEmut	7	131.43	27.97	8.94×10^{-99}	0.14	0.14	0.18

Supplementary Table 12. Pairwise associations in all samples from QUASAR2 and Australia combined.
Red shading denotes a negative association, green shading a positive association.

			MSI			*KRAS*			*BRAF*			*TP53*		
		Total	MSS	MSI+	P	Wt	Mut	P	Wt	Mut	P	Wt	Mut	P
MSI (%)	MSI-	1488 (86)	-	-		-	-		-	-		-	-	
	MSI+	244 (14)	-	-		-	-		-	-		-	-	
KRAS (%)	Wt	1102 (64)	894 (60)	208 (85)	2×10^{-15}	-	-		-	-		-	-	
	Mut	630 (36)	594 (40)	36 (15)		-	-		-	-		-	-	
BRAF (%)	Wt	1504 (87)	1376 (92)	128 (52)	7×10^{-49}	880 (80)	624 (99)	2×10^{-39}	-	-		-	-	
	Mut	228 (13)	112 (8)	116 (48)		222 (20)	6 (1)		-	-		-	-	
TP53 (%)	Wt	473 (41)	361 (36)	112 (72)	1×10^{-16}	274 (39)	199 (45)	0.031	388 (39)	85 (56)	9×10^{-5}	-	-	
	Mut	674 (59)	630 (64)	44 (28)		433 (61)	241 (55)		607 (61)	67 (44)		-	-	
T (%)	T1-3	1197 (69)	1033 (69)	164 (67)	0.5	781 (71)	416 (66)	0.04	1056 (70)	141 (62)	0.014	324 (69)	494 (73)	0.085
	T4	535 (31)	455 (31)	80 (33)		321 (29)	214 (34)		448 (30)	87 (38)		149 (31)	180 (27)	
N (%)	N0	637 (37)	504 (34)	133 (55)	1×10^{-9}	425 (39)	212 (34)	0.043	552 (37)	85 (37)	0.88	188 (40)	217 (32)	0.01
	N1-2	1095 (63)	984 (66)	111 (45)		677 (61)	418 (66)		952 (63)	143 (63)		285 (60)	457 (68)	
Gender (%)	Male	979 (57)	874 (59)	105 (43)	5×10^{-6}	630 (57)	349 (55)	0.48	885 (59)	94 (41)	7×10^{-7}	245 (52)	372 (55)	0.28
	Female	753 (43)	614 (41)	139 (57)		472 (43)	281 (45)		619 (41)	134 (59)		228 (48)	302 (45)	
Location (%)	Proximal	676 (41)	485 (35)	191 (83)	5×10^{-45}	413 (40)	263 (44)	0.15	502 (35)	174 (80)	7×10^{-36}	227 (49)	225 (34)	9×10^{-7}
	Distal	959 (59)	921 (65)	38 (17)		620 (60)	339 (56)		916 (65)	43 (20)		234 (51)	428 (66)	
Differentiation (%)	Well/Mod	1361 (82)	1230 (87)	131 (55)	1×10^{-28}	839 (80)	522 (87)	7×10^{-5}	1230 (86)	131 (60)	1×10^{-17}	365 (80)	537 (83)	0.34
	Poor	291 (18)	185 (13)	106 (45)		215 (20)	76 (13)		205 (14)	86 (40)		90 (20)	113 (17)	
Age (SE)	Mean	65.2 (0.26)	65.0 (0.27)	66.4 (0.73)	0.03	64.9 (0.33)	65.8 (0.42)	0.061	64.7 (0.28)	68.4 (0.63)	5×10^{-7}	66.4 (0.51)	65.3 (0.43)	0.048

Supplementary Table 13. Stepwise multivariable regression analysis searching for associations between clinico-pathological-molecular variables.
and * predicted perfect or near-perfect success and failure respectively and hence were formally dropped from the analysis, but are shown here for completeness' sake.

Outcome	Tumours	Variable 1	Variable 2	Variable 3	Variable 4	Variable 5	Variable 6	Variable 7	Variable 8
APC	426	*BRAF*<br>OR=0.06, P=6.02x10^{-13}	*TP53*<br>OR=1.77, P=0.023	CIN<br>OR=0.53, P=0.034	Left vs right location<br>OR=1.66, P=0.041	T4 vs T1/2/3<br>OR=0.61, P=0.044	POLE#		
TP53	449	MSI<br>OR=0.13, P=4.88x10^{-6}	*ATM*<br>OR=0.17, P=0.001	CIN<br>OR=2.14, P=0.002	*NF1*<br>OR=0.23, P=0.019	*GNAS**			
KRAS	479	*BRAF*<br>OR=0.01, P=1.5x10^{-5}	Left v Right location<br>OR=0.46, P=0.001	F v M gender<br>OR=1.63, P=0.018	MSI<br>OR=0.37, P=0.020	*PIK3CA*<br>OR=1.81, P=0.021	*NRAS**		
PIK3CA	511	*KRAS*<br>OR=1.95, P=0.004	*FBXW7*<br>OR=2.05, P=0.019	*TP53*<br>OR=0.63, P=0.049					
FBXW7	484	Grade<br>OR=2.92, P=2.07x10^{-4}	*PIK3CA*<br>OR=2.35, P=0.008	*NRAS*<br>OR=7.34, P=0.012	*TP53*<br>OR=1.96, P=0.031	*CTNNB1**			
BRAF	479	*APC*<br>OR=0.11, P=7.47x10^{-9}	*KRAS*<br>OR=0.02, P=8.50x10^{-5}	Left v Right location<br>OR=0.24, P=9.23x10^{-5}	F v M gender<br>OR=2.47, P=0.009	MSI<br>OR=2.27, P=0.045	*CTNNB1* & POLE*		
SMAD4	484	*BRAF*<br>OR=3.62, P=0.001	Grade<br>OR=0.49, P=0.044	*CTNNB1, GNAS, NRAS, NF1* & POLE*					
GNAS	511	*TP53*<br>OR=0.09, P=0.023	*PTEN, NRAS, ATM*, POLE, gender & location*						
CTNNB1	484	MSI<br>OR=10.44, P=0.002	*KRAS*<br>OR=8.06, P=0.010	*APC*<br>OR=0.19, P=0.029	Grade<br>OR=3.54, P=0.036	*NF1, GNAS, SMAD4, BRAF, FBXW7, PTEN, NRAS, ATM* & POLE*			
PTEN	511	*POLE*<br>OR=24.45, P=7.50x10^{-5}	*CTNNB1, GNAS, SMAD4, NRAS* & MSI*						
NRAS	511	*NF1*<br>OR=19.52, P=1.28x10^{-4}	*KRAS, PTEN, CTNNB1, GNAS, SMAD4, ATM*, MSI & POLE *						
ATM	511	*TP53*<br>OR=0.27, P=0.006	POLE<br>OR=10.13, P=0.010	*NRAS, CTNNB1* & *GNAS* *					
NF1	479	*POLE*<br>OR=30.25, P=3.5x10^{-5}	*NRAS*<br>OR=24.2, P=5.12x10^{-5}	Left v Right location<br>OR=0.28, P=0.045	*CTNNB1* & *SMAD4* *				
MSI	416	CIN<br>OR=0.05, P=6.04x10^{-7}	*BRAF*<br>OR=11.02, P=1.36x10^{-5}	*TP53*<br>OR=0.07, P=2.55x10^{-5}	Left v Right location<br>OR=0.20, P=0.005	*CTNNB1*<br>OR=20.77, P=0.007	Grade<br>OR=3.75, P=0.007	*ATM*<br>OR=0.10, P=0.037	*PTEN, NRAS* & POLE *
POLE	511	Age<br>OR=0.83, P=1.18x10^{-4}	*NF1*<br>OR=41.75, P=0.002	*FBXW7*<br>OR=13.10, P=0.015	MSI, CIN, *APC, CTNNB1, BRAF, SMAD4* & *GNAS* *				
CIN	449	MSI<br>OR=0.08, P=2.23x10^{-9}	*TP53*<br>OR=2.06, P=0.003	*PTEN*<br>OR=0.21, P=0.012	*PIK3CA*<br>OR=0.56, P=0.042	POLE *			
T4 vs T1/2/3	471	Stage III v II<br>OR=0.47, P=1.45x10^{-4}	Left v Right location<br>OR=0.62, P=0.013	POLE *					
Stage III vs II	511	T4 vs T1/2/3<br>OR=0.47, P=1.2x10^{-4}	Age<br>OR=1.03, P=0.011						
F v M gender	511	*BRAF*<br>OR=3.17, P=2.7x10^{-5}	*KRAS*<br>OR=1.63, P=0.012	*GNAS*#					
Distal v Proximal location	479	*BRAF*<br>OR=0.20, P=3.66x10^{-6}	MSI<br>OR=0.18, P=5.80x10^{-6}	*KRAS*<br>OR=0.43, P=2.00x10^{-4}	*PTEN*<br>OR=0.19, P=0.008	*APC*<br>OR=1.68, P=0.029			
Age	511	POLE<br>OR=1.6x10^{-9}, P=2.8x10^{-8}	Stage II v III<br>OR=6.50, P=0.036						
Differentiation	484	MSI<br>OR=1.36, P=1.5x10^{-5}	*FBXW7*<br>OR=1.27, P=0.0004	*CTNNB1*<br>OR=1.40, P=0.023					

Supplementary Table 14. Proportion of putative sub-clonal mutations by genes.

Gene	Clonal mutations in gene (%)	Sub-clonal mutations in gene (%)	Clonal mutations in other 12 genes (%)	Sub-clonal mutations in other 12 genes (%)	$P_{Fisher's\ exact}$
APC	447 (95)	22 (5)	856 (93)	62 (7)	0.15
ATM	17 (74)	6 (26)	1286 (94)	78 (6)	0.002
BRAF	79 (100)	0	1224 (94)	84 (6)	0.012
CTNNB1	12 (86)	2 (14)	1291 (94)	82 (6)	0.21
FBXW7	60 (86)	10 (14)	1243 (94)	74 (6)	0.08
GNAS	8 (100)	0	1295 (94)	84 (6)	1
KRAS	201 (98)	5 (2)	1102 (93)	79 (7)	0.017
NF1	17 (94)	1 (6)	1286 (94)	83 (6)	0.24
NRAS	8 (100)	0	1295 (94)	84 (6)	1
PIK3CA	86 (85)	15 (15)	1217 (95)	69 (5)	0.001
PTEN	20 (95)	1 (5)	1283 (94)	83 (6)	1
SMAD4	29 (85)	5 (15)	1274 (94)	79 (6)	0.05
TP53	319 (95)	17 (5)	984 (94)	67 (6)	0.43

Supplementary Table 15. Associations of individual molecular markers with RFS in QUASAR2 (univariable analysis adjusted by T, N and study arm; N=511).

	RFS multivariable per gene		
	HR	95% CI	P
**Sub-clonal mutations**	0.76	0.45-1.31	0.327
**Mutation burden (quartiles)**	0.87	0.75-1.00	0.055
**MSI**	0.73	0.42-1.28	0.271
**CIN**	1.39	0.91-2.10	0.123
**APC**	0.80	0.57-1.11	0.180
**TP53**	1.53	1.08-2.18	0.018
**KRAS**	1.48	1.07-2.05	0.018
**PIK3CA**	0.80	0.52-1.24	0.316
**BRAF**	1.42	0.94-2.13	0.093
**FBXW7**	0.98	0.59-1.63	0.936
**SMAD4**	0.68	0.33-1.39	0.288
**ATM**	0.77	0.31-1.87	0.560
**NF1**	0.39	0.10-1.56	0.182
**PTEN**	1.12	0.45-2.75	0.810
**CTNNB1**	0.63	0.20-1.99	0.436
**GNAS**	2.19	0.89-5.35	0.087
**NRAS**	0.46	0.06-3.35	0.445

Supplementary Table 16. Associations of combinations of co-varying molecular markers with RFS in QUASAR2 (univariable analysis adjusted by T, N and study arm; N=511).

The *TP53-ATM* association was driven almost entirely by an association between *TP53* and poor prognosis.

	RFS multivariable		
	HR	95% CI	P
**NRAS/NF1**	0.44	0.14-1.40	0.166
**ATM/PTEN**	0.83	0.42-1.64	0.595
**BRAF/SMAD4**	1.32	0.90-1.93	0.154
**TP53/ATM**	1.41	0.99-2.02	0.058

Supplementary Table 17. Associations of mutation burden with clinico-pathological variables in QUASAR2 and Australian cohorts combined (MSI-/POLE non-pathogenic cancers only, multivariable linear regression; N**=638).**

Whilst high mutation burden in the combined sample set was associated with proximal cancer location and older age, addition of location and age to the prognostic model did not affect the associations of any of our prognostic markers in any important way (data not shown).

Mutation burden in MSI-/POLEwt	OR	95%CI	P
**Male vs Female**	1.05	0-88-1.26	0.575
**Proximal v Distal**	1.32	1.08-1.59	0.006
**Poor vs Well/Moderate**	0.97	0.75-1.27	0.841
**Age**	1.01	1.00-1.02	0.023
**T4 v T123**	0.90	0.75-1.09	0.28
**N+ v N0**	0.98	0.81-1.19	0.847

Supplementary Table 18. Associations between clinico-pathological-molecular variables and relapse-free survival broken down by stage.

(a) Stage II. N=276 univariable); N=266 multivariable); N=209 (MSI-/Non-pathogenic POLE multivariable).

Stage II	All cases univariable			All cases multivariable			MSI- & Non-pathogenic POLE multivariable		
	HR	95%CI	P	HR	95%CI	P	HR	95%CI	P
KRAS mutation	1.38	0.87-2.19	0.17	1.77	1.03-3.05	0.040	1.98	1.09-3.58	0.024
BRAF mutation	0.94	0.47-1.89	0.87	1.77	0.74-4.21	0.197	1.58	0.44-5.65	0.478
TP53 mutation	0.98	0.62-1.56	0.96	1.13	0.67-1.89	0.646	1.15	0.65-2.02	0.635
Mutation burden (quartiles)	0.88	0.72-1.07	0.19	0.86	0.67-1.11	0.245	0.88	0.68-1.13	0.299
MSI	0.67	0.35-1.26	0.21	0.79	0.34-1.85	0.590	-		
Cohort/treatment Q2 cap	Ref.			Ref.			Ref.		
Cohort/treatment Q2 bev+cap	2.17	1.07-4.42	0.032	2.18	1.07-4.44	0.032	2.71	1.20-6.10	0.017
Cohort/treatment Australia no chemo	2.66	1.32-5.35	0.006	3.17	1.41-7.14	0.005	4.70	1.83-12.0	0.001
Cohort/treatment Australia chemo	3.45	1.46-8.12	0.005	4.53	1.84-11.2	0.001	6.87	2.51-18.8	$2x10^{-4}$
Radiotherapy (yes vs no)	3.99	0.98-16.3	0.054	2.78	0.60-12.8	0.190	2.66	0.57-12.5	0.215
T4 v T123	1.22	0.70-1.80	0.63	1.43	0.80-2.54	0.231	1.83	0.96-3.47	0.065

(b) Stage III. N=531 univariable); N=499 multivariable); N=444 (MSI-/Non-pathogenic POLE multivariable).

Stage III	All cases univariable			All cases multivariable			MSI- & Non-pathogenic POLE multivariable		
	HR	95%CI	P	HR	95%CI	P	HR	95%CI	P
**KRAS mutation**	1.42	1.07-1.88	0.015	1.81	1.30-2.53	$4x10^{-4}$	1.91	1.36-2.68	$2x10^{-4}$
**BRAF mutation**	1.36	0.93-1.99	0.12	2.53	1.60-3.99	$7x10^{-5}$	2.65	1.65-4.24	$5x10^{-5}$
**TP53 mutation**	1.41	1.04-1.91	0.026	1.90	1.34-2.68	$3x10^{-5}$	1.88	1.31-2.70	0.001
**Mutation burden (quartiles)**	0.81	0.71-0.92	0.002	0.77	0.66-0.90	0.001	0.81	0.70-0.93	0.003
**MSI**	0.58	0.33-1.05	0.072	0.76	0.38-1.54	0.449	-		
**Cohort/treatment Q2 cap**	Ref.			Ref.			Ref.		
**Cohort/treatment Q2 bev+cap**	1.25	0.85-1.82	0.26	1.25	0.85-1.84	0.254	1.29	0.86-1.92	0.213
**Cohort/treatment Australia no chemo**	2.68	1.64-4.38	$8x10^{-5}$	3.71	2.17-6.32	$1x10^{-6}$	4.01	2.30-7.00	$1x10^{-6}$
**Cohort/treatment Australia chemo**	1.68	1.14-2.47	0.009	1.43	0.93-2.20	0.106	1.49	0.95-2.32	0.083
**Radiotherapy (yes vs no)**	1.02	0.33-3.21	0.970	1.11	0.34-3.60	0.868	1.04	0.32-3.39	0.949
**T4 v T123**	2.26	1.71-2.99	$1x10^{-8}$	2.50	1.85-3.36	$2x10^{-9}$	2.59	1.91-3.53	$1x10^{-9}$

Supplementary Table 19. Associations between clinico-pathological-molecular variables and relapse-free survival broken down by tumour location.

(a) Proximal colon. N=318 (univariable); N=310 (multivariable); N=219 (MSI-/Non-pathogenic POLE).

Proximal colon	All cases univariable			All cases multivariable			MSI- & Non-pathogenic POLE multivariable		
	HR	95%CI	P	HR	95%CI	p	HR	95%CI	p
**KRAS mutation**	1.12	0.77-1.62	0.56	1.37	0.86-2.17	0.182	1.68	1.01-2.79	0.046
**BRAF mutation**	1.35	0.90-2.02	0.15	1.99	1.20-3.31	0.008	2.32	1.28-4.20	0.006
**TP53 mutation**	1.66	1.14-2.42	0.008	1.50	0.99-2.28	0.056	1.57	0.98-2.53	0.061
**Mutation burden (quartiles)**	0.75	0.64-0.88	0.0004	0.80	0.66-0.97	0.024	0.79	0.66-0.96	0.017
**MSI**	0.56	0.35-0.89	0.015	0.80	0.43-1.52	0.499	-		
**Cohort/treatment Q2 cap**	Ref.			Ref.			Ref.		
**Cohort/treatment Q2 bev+cap**	1.63	0.99-2.68	0.054	1.58	0.96-2.60	0.074	1.77	1.01-3.11	0.046
**Cohort/treatment Australia no chemo**	1.59	0.88-2.87	0.12	3.01	1.54-5.88	0.001	3.58	1.61-7.97	0.002
**Cohort/treatment Australia chemo**	2.16	1.26-3.71	0.005	2.04	1.16-3.56	0.013	2.17	1.16-4.06	0.015
**Radiotherapy (yes vs no)**	-			-			-		
**T4 v T123**	1.78	1.23-2.57	0.002	2.02	1.37-2.98	0.0004	2.22	1.43-3.43	0.0004
**N+ vs N0**	2.19	1.44-3.34	0.0003	2.28	1.43-3.64	0.001	2.67	1.51-4.72	0.001

(b) Distal colon. N=293 univariable); N=286 multivariable); N=268 (MSI-/Non-pathogenic POLE).

Distal colon	All cases univariable			All cases multivariable			MSI- & Non-pathogenic POLE multivariable		
	HR	95%CI	P	HR	95%CI	p	HR	95%CI	p
**KRAS mutation**	2.02	1.31-3.11	0.002	2.46	1.53-3.98	0.0002	2.43	1.50-3.95	0.0003
**BRAF mutation**	0.75	0.27-2.04	0.566	1.46	0.51-4.13	0.478	1.55	0.55-4.36	0.403
**TP53 mutation**	1.29	0.80-2.06	0.295	2.03	1.21-3.41	0.008	2.00	1.18-3.39	0.01
**Mutation burden (quartiles)**	0.88	0.71-1.08	0.212	0.87	0.69-1.09	0.222	0.96	0.77-1.18	0.673
**MSI**	0.21	0.029-1.48	0.116	0.22	0.028-1.67	0.142	-		
**Cohort/treatment Q2 cap**	Ref.			Ref.			Ref.		
**Cohort/treatment Q2 bev+cap**	1.53	0.84-2.78	0.167	1.78	0.96-3.32	0.069	1.93	1.02-3.65	0.044
**Cohort/treatment Australia no chemo**	1.63	0.77-3.45	0.200	4.51	1.50-8.19	0.004	3.85	1.63-9.12	0.002
**Cohort/treatment Australia chemo**	2.30	1.21-4.38	0.011	2.94	1.48-5.84	0.002	3.19	1.58-6.44	0.001
**Radiotherapy (yes vs no)**	-			-			-		
**T4 v T123**	2.14	1.39-3.30	0.001	2.53	1.58-4.05	0.0001	2.71	1.67-4.37	0.00004
**N+ vs N0**	1.02	0.64-1.62	0.934	1.09	0.64-1.85	0.746	1.05	0.62-1.78	0.851

(c) Rectum. N=180 (univariable); N=153 (multivariable); N=150 (MSI-/Non-pathogenic POLE).

Rectum	All cases univariable			All cases multivariable			MSI- & Non-pathogenic POLE multivariable		
	HR	95%CI	P	HR	95%CI	p	HR	95%CI	p
**KRAS mutation**	1.32	0.82-2.15	0.253	1.63	0.86-3.08	0.131	1.55	0.81-2.96	0.182
**BRAF mutation**	1.34	0.33-5.47	0.684	1.75	0.37-8.21	0.476	1.51	0.33-7.01	0.598
**TP53 mutation**	0.94	0.57-1.57	0.82	1.91	0.94-3.88	0.074	1.69	0.84-3.43	0.144
**Mutation burden (quartiles)**	0.81	0.63-1.06	0.122	0.74	0.54-1.01	0.061	0.84	0.64-1.10	0.2
**MSI**	0.71	0.10-5.14	0.738	2.57	0.29-22.69	0.395	-		
**Cohort/treatment Q2 cap**	Ref.			Ref.			Ref.		
**Cohort/treatment Q2 bev+cap**	1.47	0.66-3.23	0.343	1.28	0.57-2.88	0.546	1.26	0.56-2.85	0.572
**Cohort/treatment Australia no chemo**	4.38	2.06-9.32	0.0001	5.23	2.24-12.22	0.0001	5.02	2.15-11.71	0.0002
**Cohort/treatment Australia chemo**	2.12	1.00-4.47	0.05	0.65	0.22-1.91	0.436	0.65	0.22-1.94	0.442
**Radiotherapy (yes vs no)**	1.66	0.66-4.19	0.282	2.47	0.85-7.18	0.097	2.41	0.82-7.09	0.109
**T4 v T123**	1.69	1.01-2.83	0.045	1.98	1.05-3.70	0.033	2.08	1.11-3.88	0.022
**N+ vs N0**	1.02	0.59-1.76	0.949	1.56	0.76-3.22	0.229	1.54	0.75-3.18	0.242

Supplementary Table 20. Associations with RFS in all samples from QUASAR2 and Australia (N=1,732) showing prognostic effect of MSI in the absence of mutation burden.

	All cases univariable			All cases multivariable		
	**HR**	**95%CI**	**P**	**HR**	**95%CI**	**P**
**KRAS mutation**	1.22	1.02-1.47	0.028	1.22	1.01-1.48	0.035
**BRAF mutation**	1.03	0.80-1.34	0.819	1.53	1.14-2.04	0.004
**MSI**	0.52	0.34-0.72	8×10^{-5}	0.45	0.31-0.64	1×10^{-5}
**Cohort/treatment Q2 cap**	Ref.			Ref.		
**Cohort/treatment (Q2 bev+cap)**	1.25	0.98-1.60	0.072	1.27	1.00-1.63	0.053
**Cohort/treatment (Aus No chemo)**	2.65	1.96-3.58	2×10^{-10}	2.79	2.06-3.78	3×10^{-11}
**Cohort/treatment (Aus Chemo)**	2	1.56-2.56	6×10^{-8}	2.01	1.57-2.59	4×10^{-8}
**T4 v T123**	2.27	1.89-2.73	1×10^{-18}	2.27	1.89-2.73	2×10^{-18}
**N+ v N0**	2.19	1.75-2.74	7×10^{-12}	2.06	1.65-2.58	3×10^{-10}

Supplementary Table 21. RFS by combinations of *KRAS, BRAF V600E* and *TP53* mutations in MSI- cancers from all QUASAR2 and Australian samples combined (N=991).

P for interaction of *TP53* with *BRAF* is 0.058 and *TP53* with *KRAS* 0.62. Results are from multivariable analysis adjusted by cohort arms as shown in Table 1(c). Mutation combinations missing from the table (KRASmut/BRAFmut) are too rare for useful analysis.

Genotype	HR	95%CI	P
**KRASwt/BRAFwt/TP53wt**	Ref.		
**KRASwt/BRAFwt/TP53mut**	1.18	0.82-1.70	0.375
**KRASwt/BRAFmut/TP53wt**	1.38	0.67-2.85	0.377
**KRASwt/BRAFmut/TP53mut**	3.08	1.88-5.03	$7x10^{-6}$
**KRASmut/BRAFwt/TP53wt**	1.43	0.97-2.12	0.069
**KRASmut/BRAFwt/TP53mut**	1.85	1.28-2.67	0.001
**T4 v T123**	2.20	1.76-2.75	$5x10^{-12}$
**N+ v N0**	2.07	1.54-2.78	$1x10^{-6}$

Supplementary Figures

Supplementary Figure 1. Study schema showing sample selection in each cohort for each type of survival analysis.

(A) QUASAR2

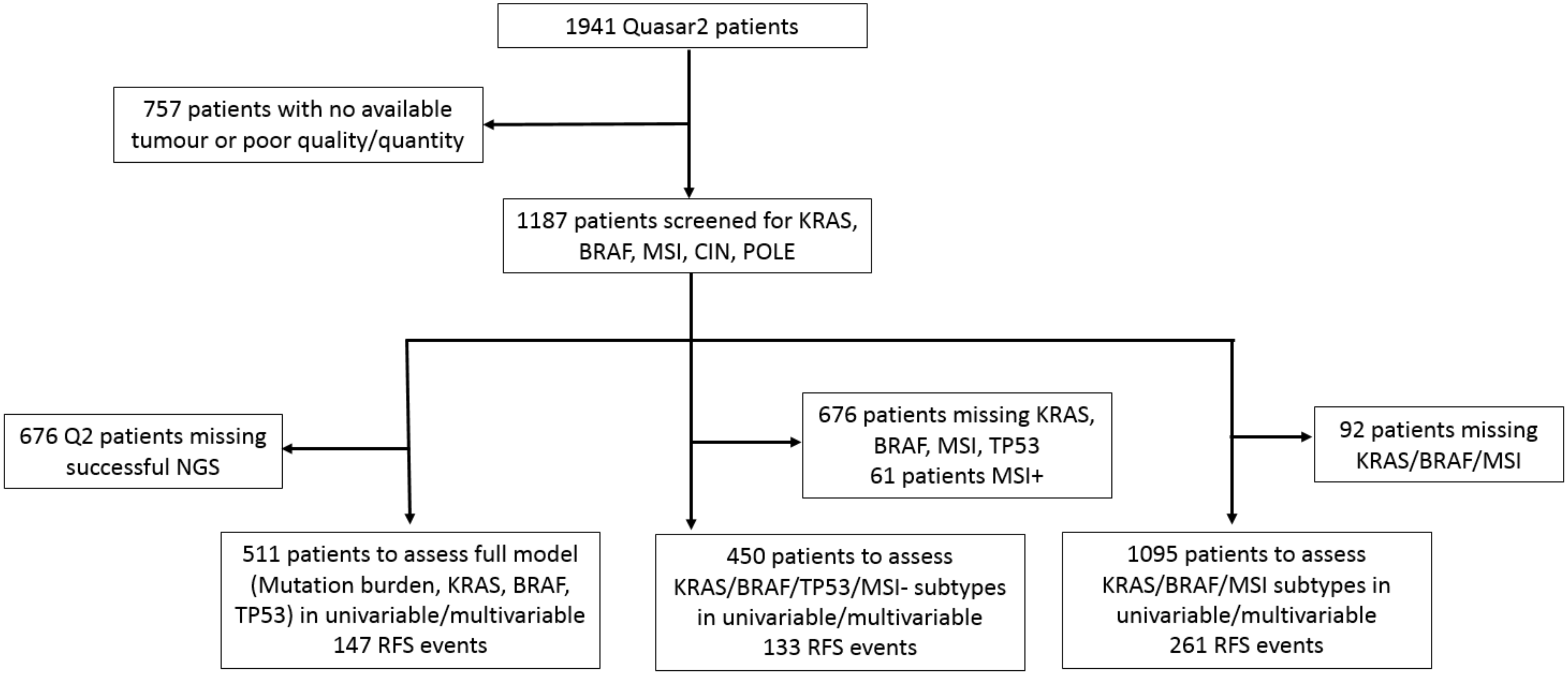

(B) Australian cohort

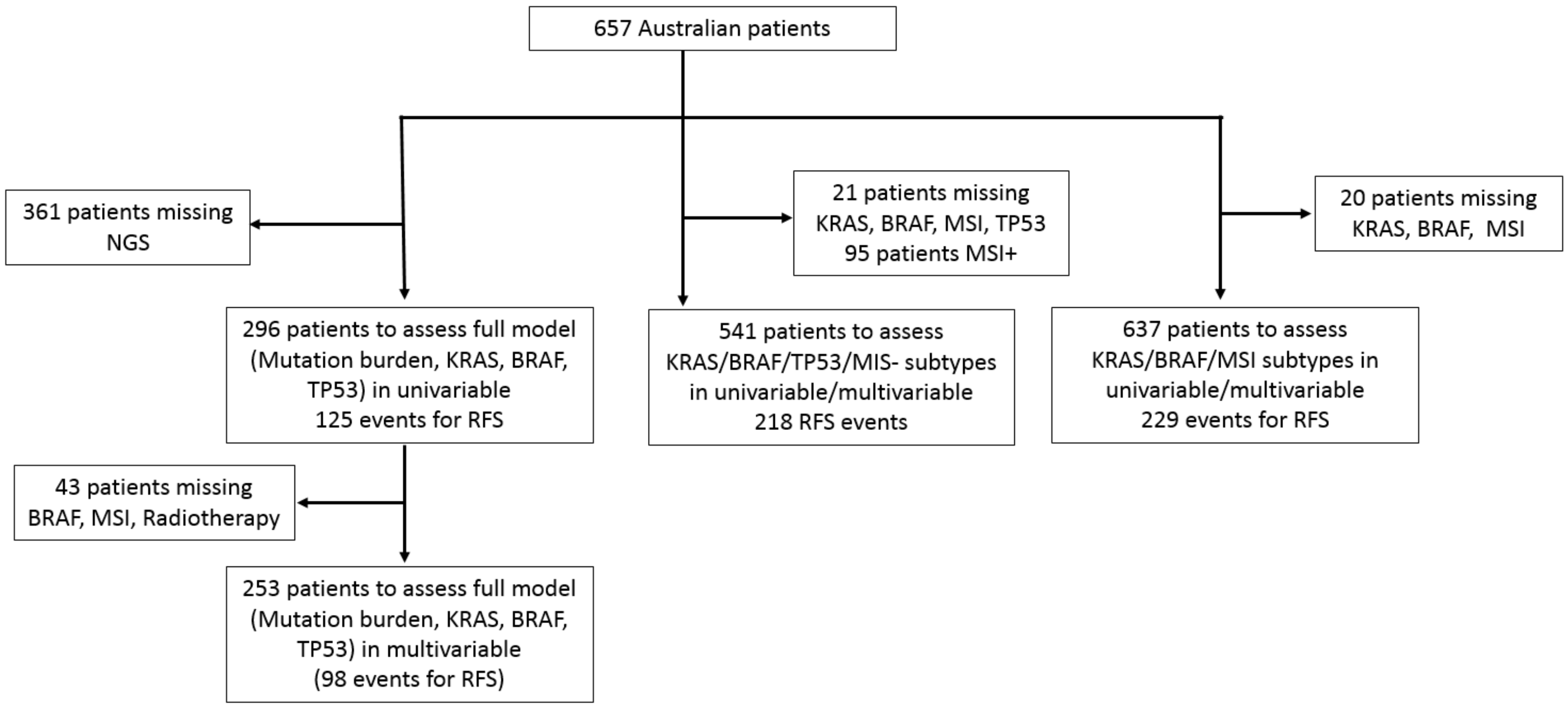

Supplementary Figure 2. Average depth of coverage on a logarithmic scale (y-axis) in all amplicons in QUASAR2 tumours and paired normal samples, sorted by tumour in increasing order (x-axis).

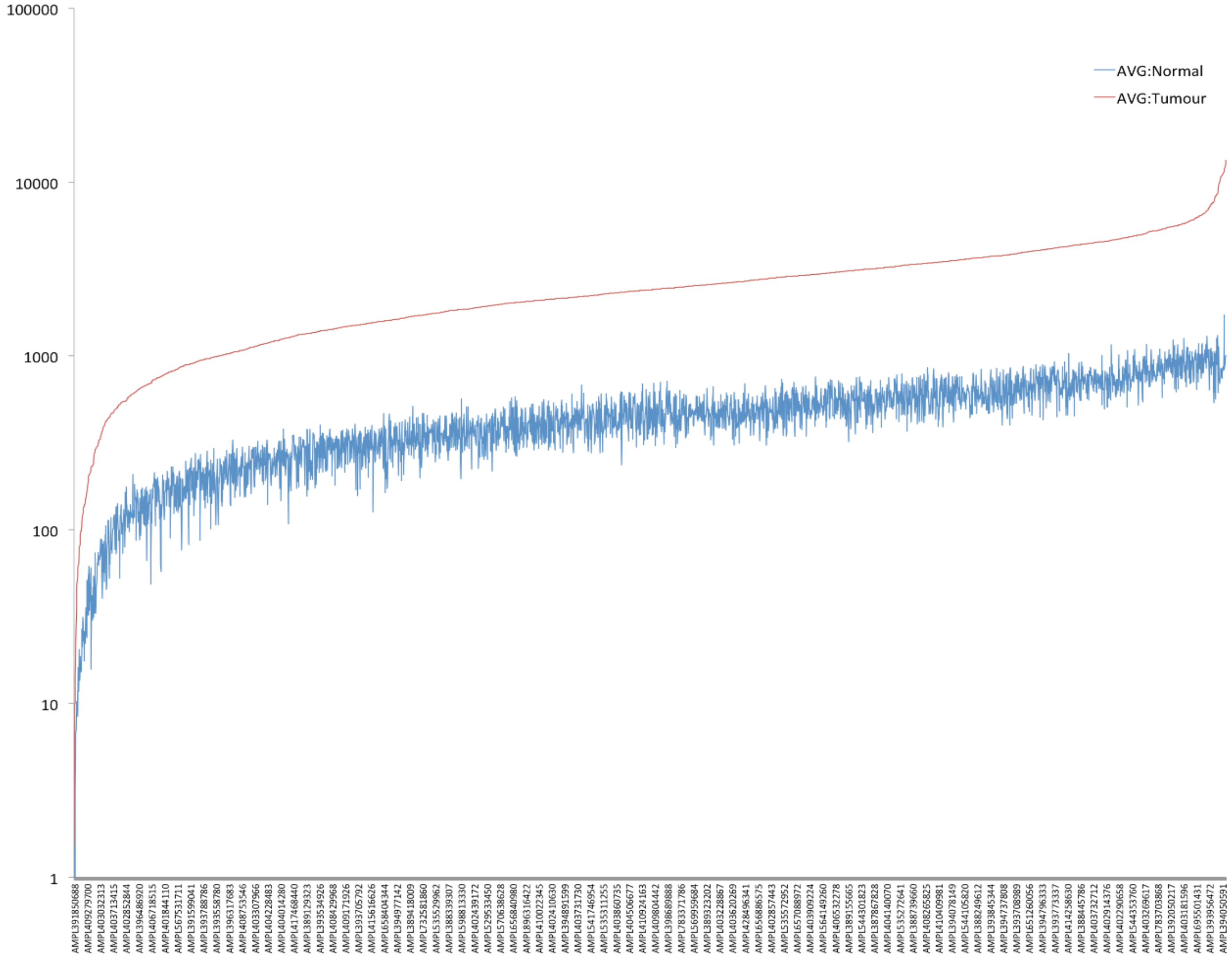

Supplementary Figure 3. ROC curve to filter in high quality somatic mutations from targeted sequencing in QUASAR2.
The corresponding model is shown in Supp. Table 8.

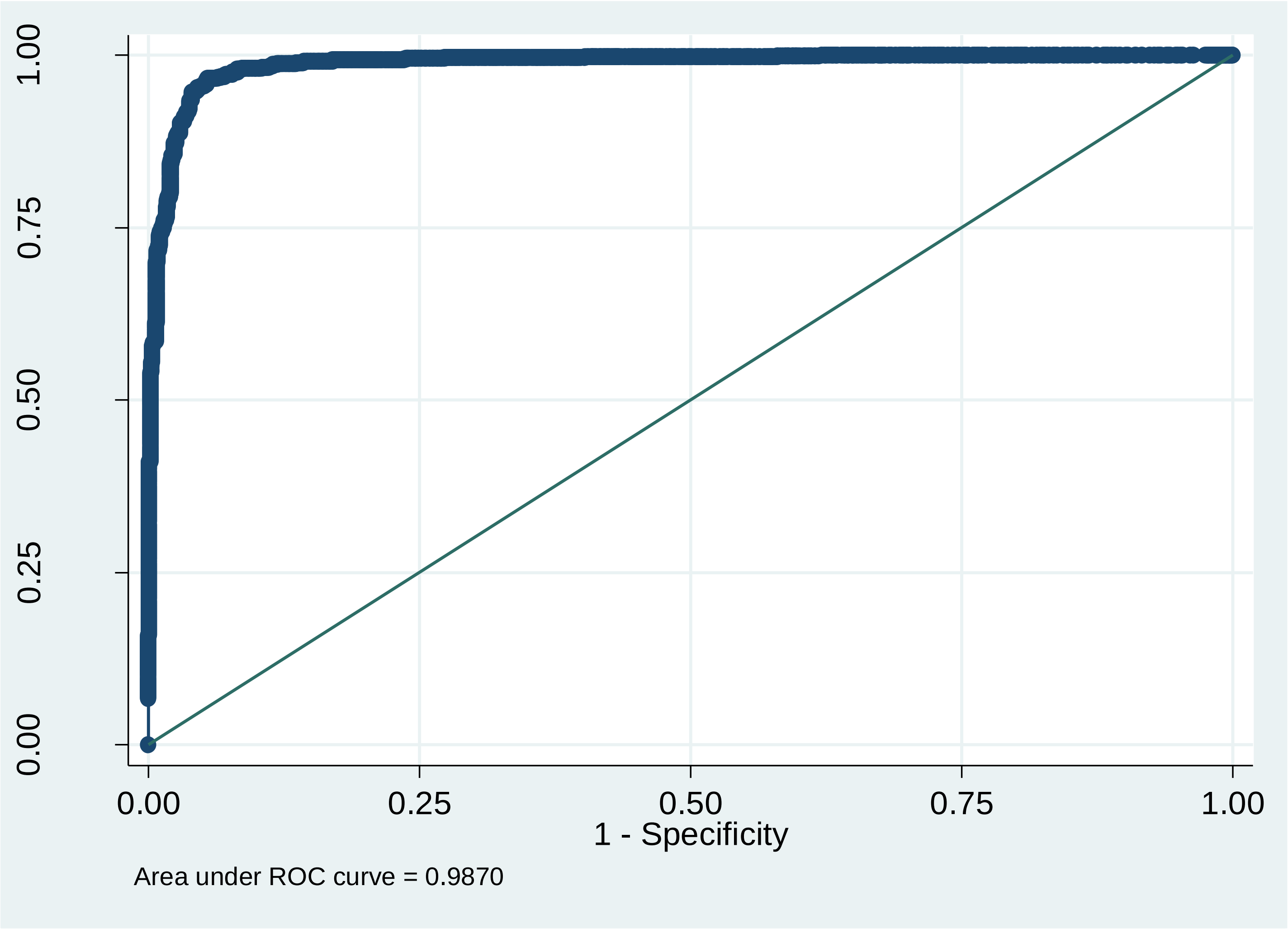

Supplementary Figure 4. Hypermutation and frequency of C>T/G>A changes in FFPE QUASAR2 and fresh frozen TCGA CRCs.
Gene contents were harmonized between the respective Ion Torrent and Exome-seq platforms. QUASAR2 tumours shown in red were excluded from further analysis due to potentially high levels of deamination.

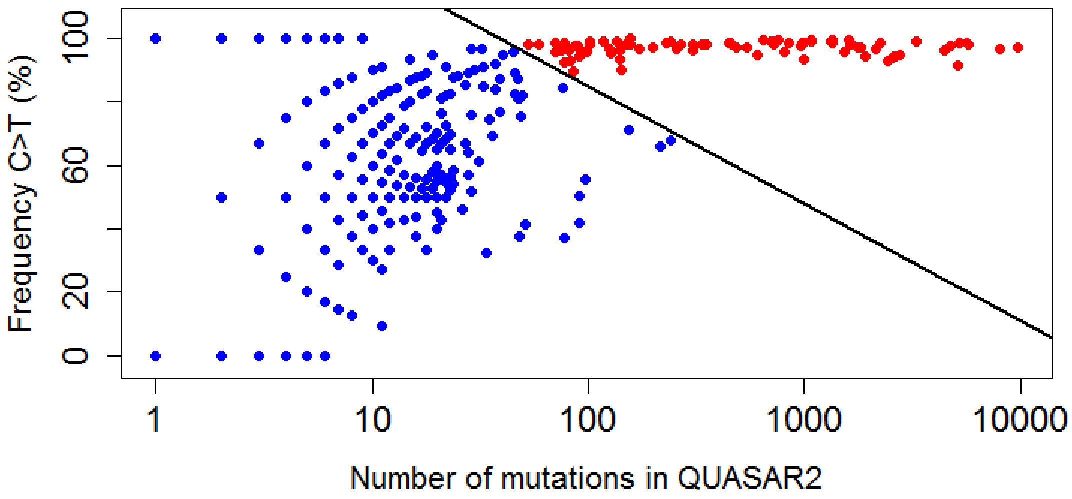

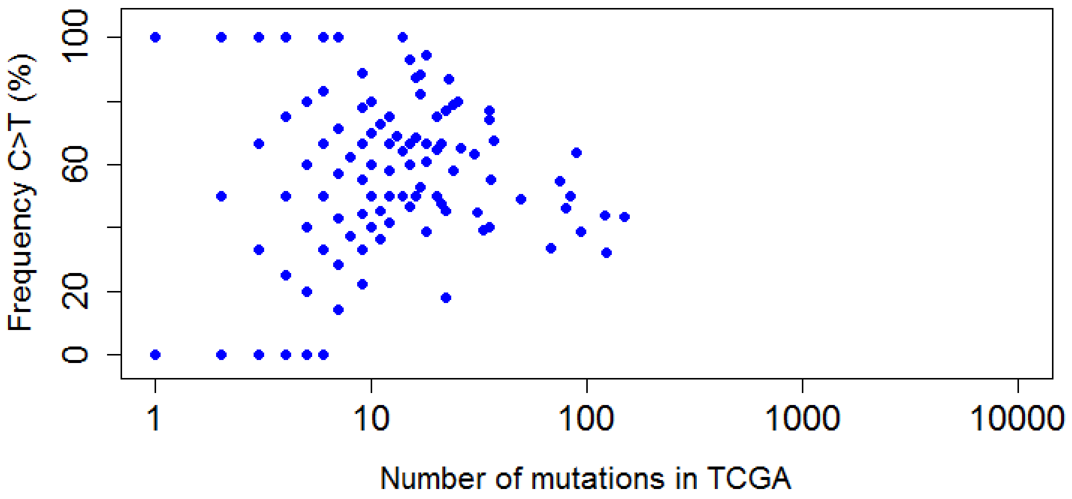

Supplementary Figure 5. Mutation spectra in QUASAR2 tumours either classified as deaminated and excluded, or non-deaminated and included, the latter shown according to genomic instability status (MSI+, MSI- (MSS) and POLE exonuclease domain mutant).
Note the predominant C>T mutation spectrum in deaminated samples differs from that in the other samples.

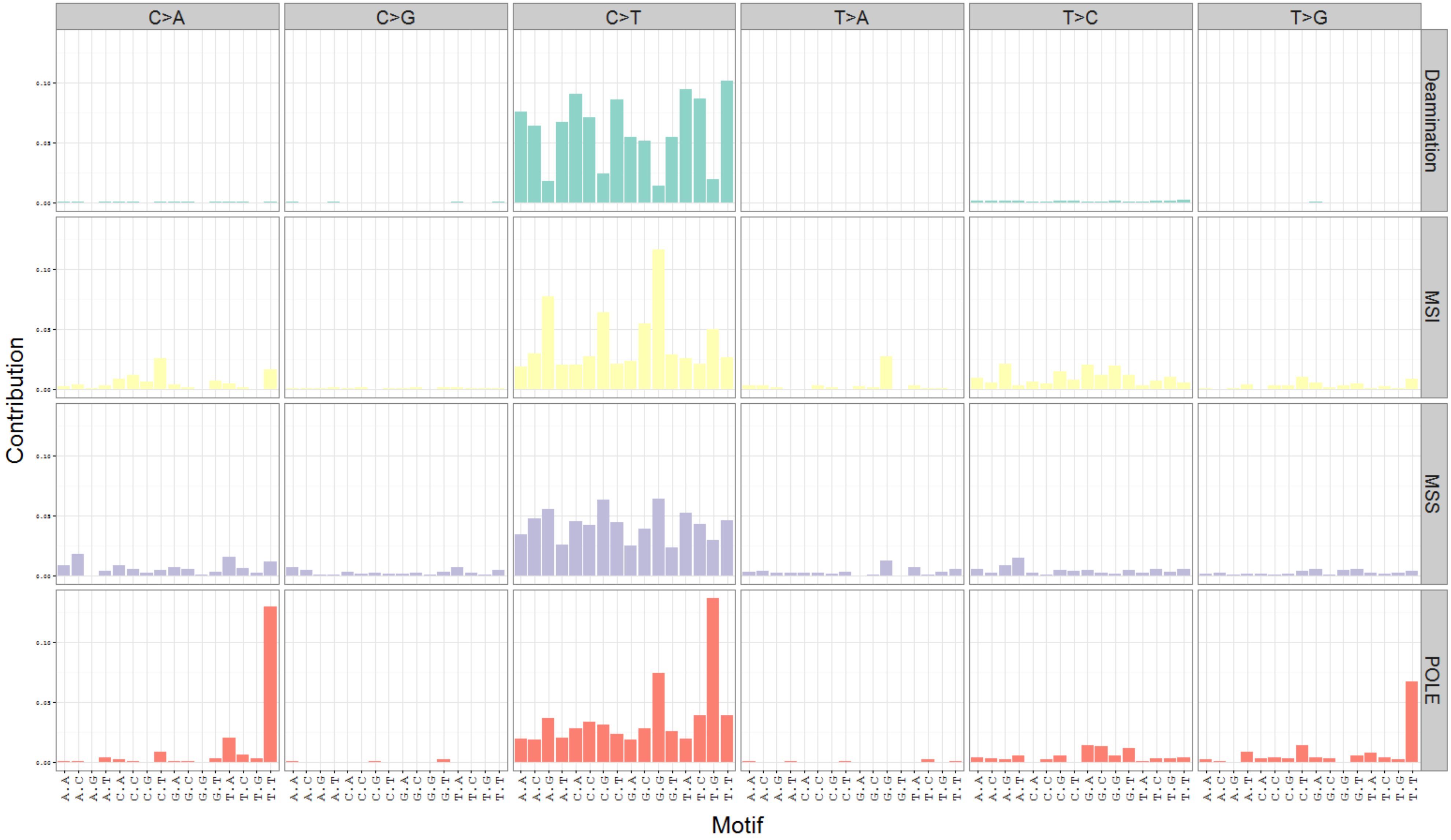

Supplementary Figure 6. QUASAR2 tumours sorted by total number of somatic mutations.
The type of genomic instability is shown in red, green and blue for MSI-/POLEwt, MSI+ and POLEmut respectively.

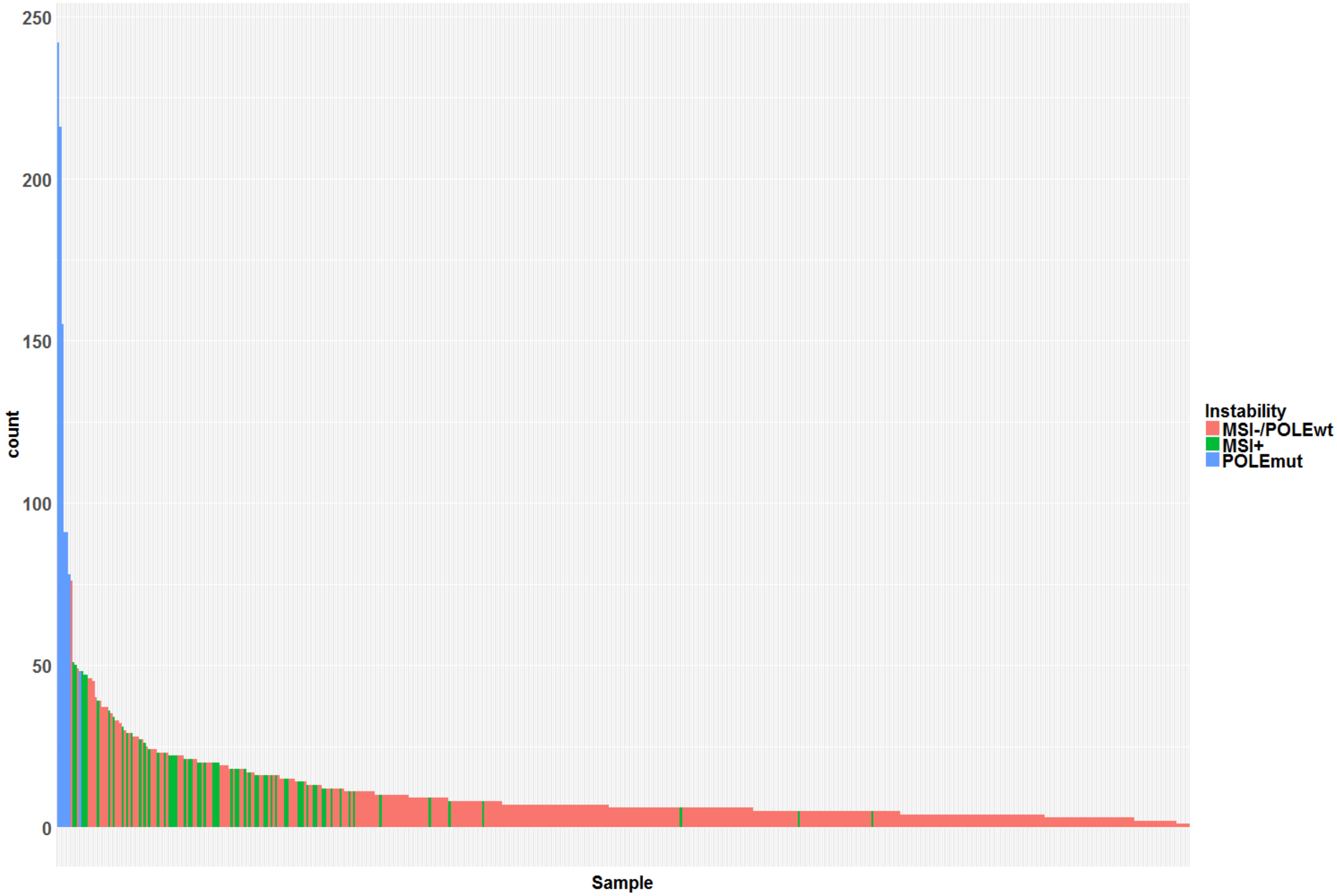

Supplementary Figure 7. Hierarchical cluster analysis by driver mutation in QUASAR2.

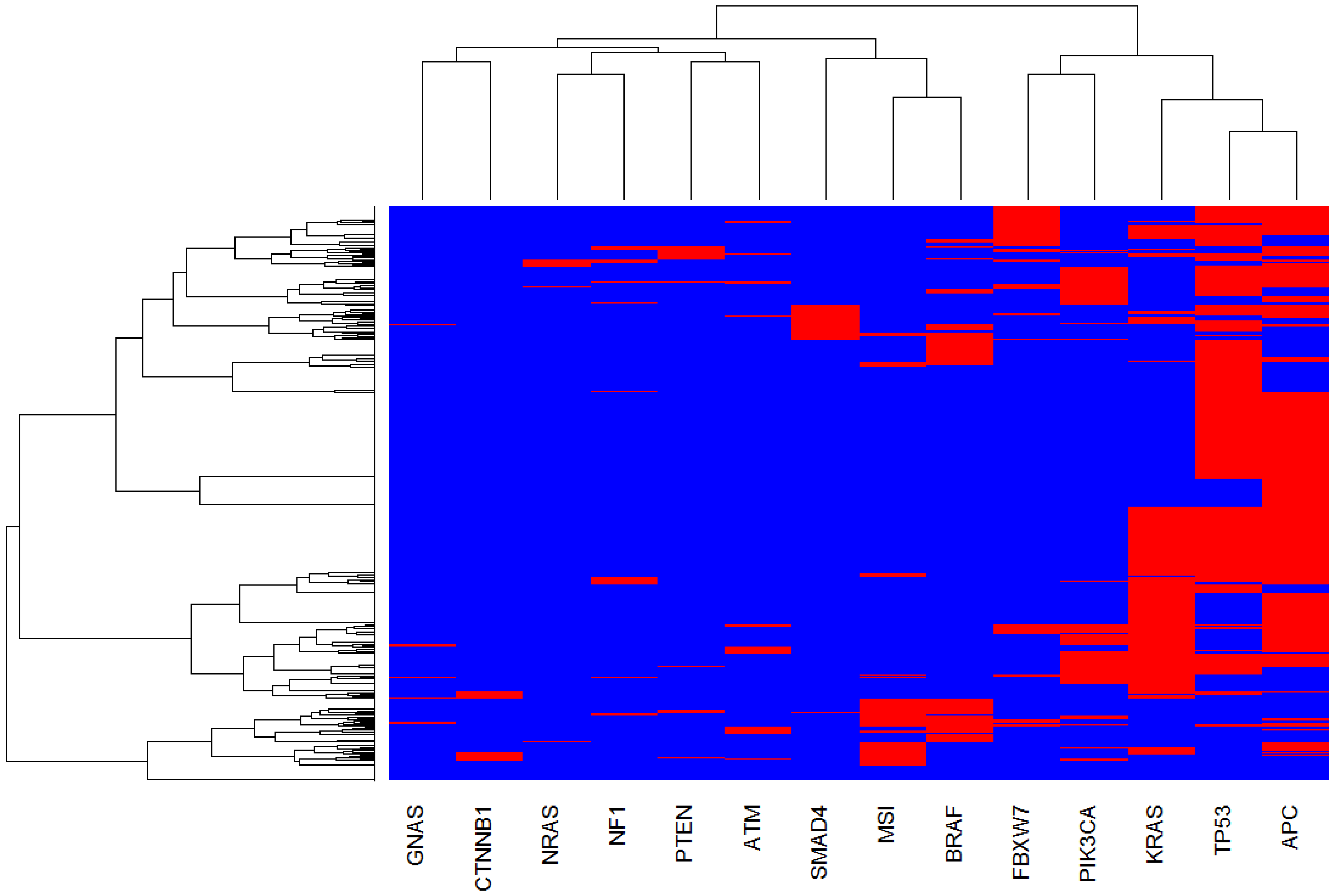

Supplementary Figure 8. Bayesian network analysis by driver mutation in QUASAR2.

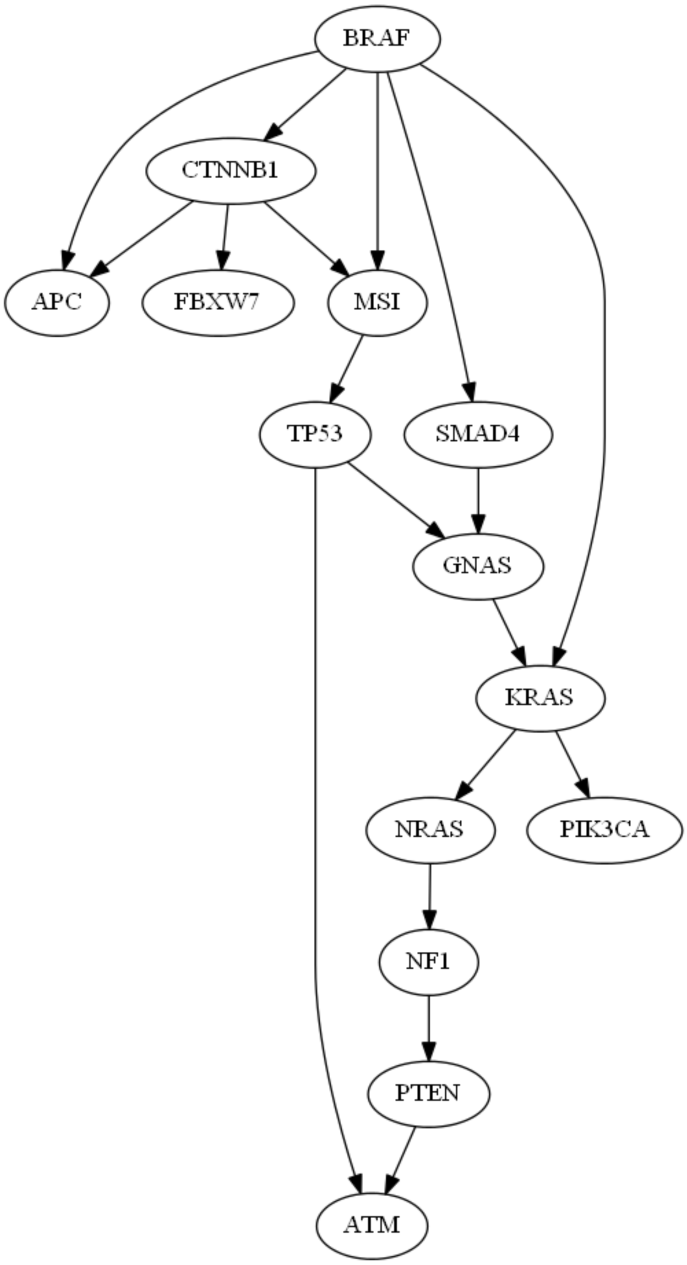

Supplementary Figure 9. Mutation spectra in Australian tumours according to genomic instability status (MSI+, MSI- (MSS) and *POLE* exonuclease domain mutant).

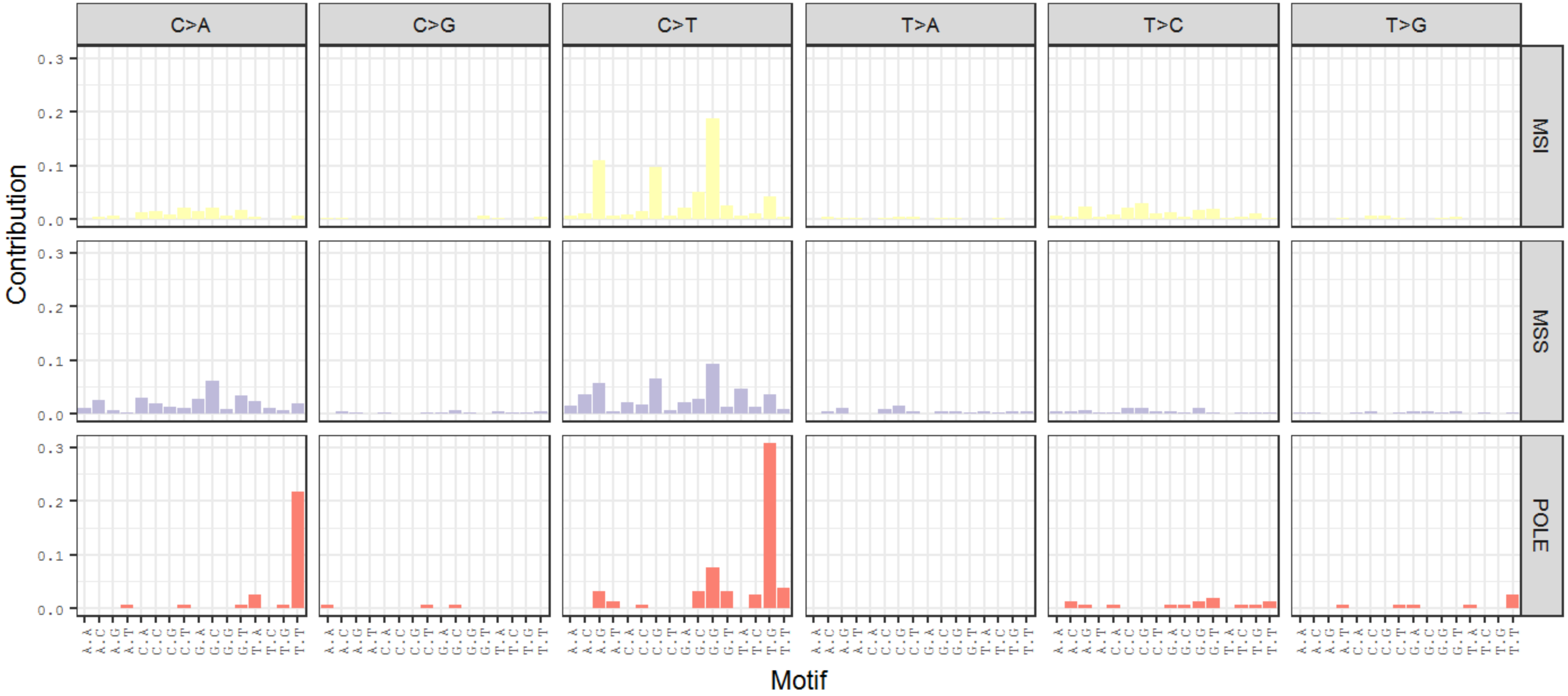

Supplementary Figure 10. Australian tumours sorted by total number of somatic mutations.
The type of genomic instability is shown in red, green and blue for MSI-/POLEwt, MSI+ and POLEmut respectively.

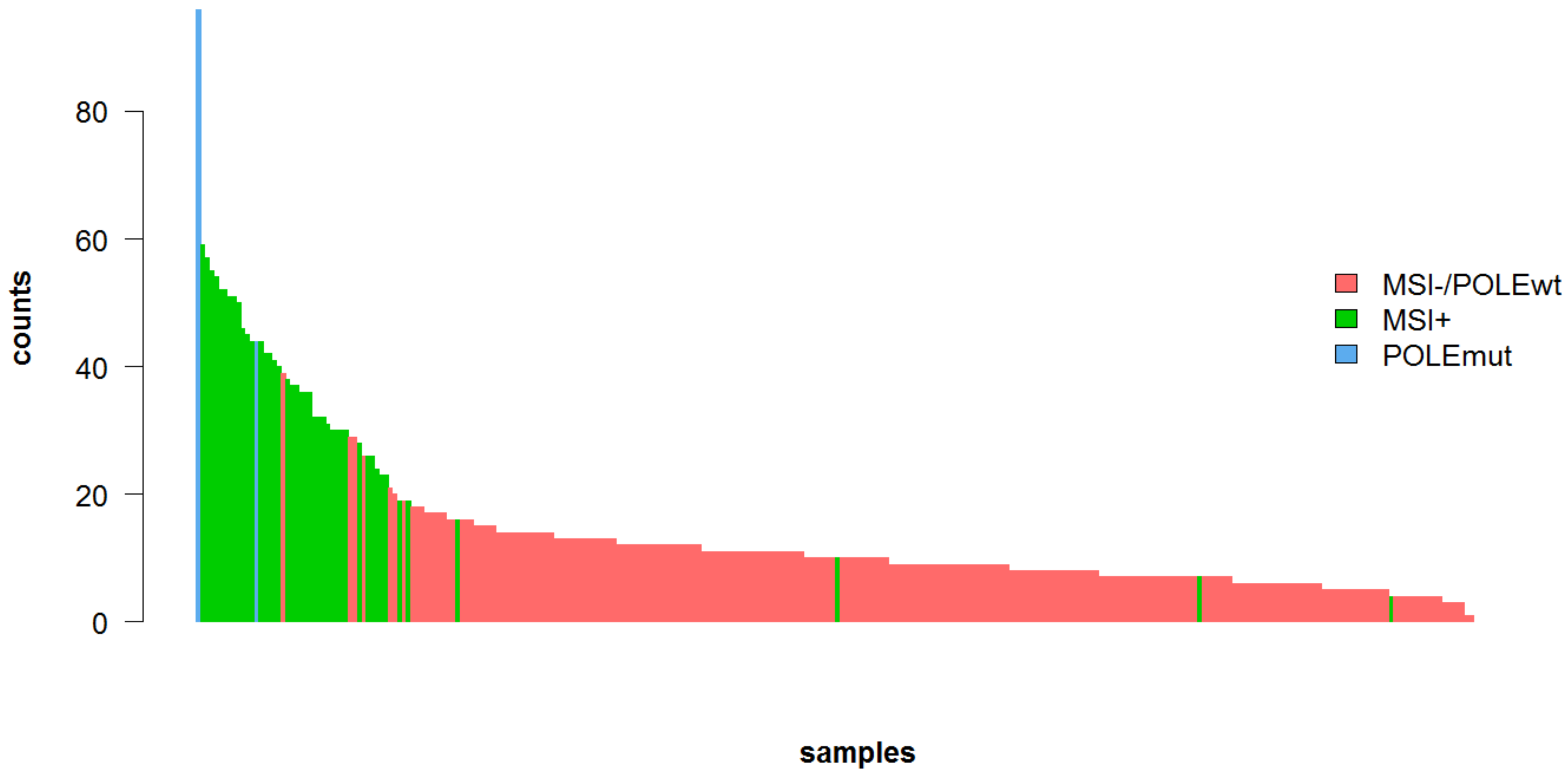

Supplementary Figure 11. Correlation between numbers of non-synonymous and coding indels with total mutations in QUASAR2 (N=511).
A fitted line is shown in blue with 95% confidence intervals shaded in grey. The plot shows that the number of non-synonymous SNVs and coding indels, our preferred measure of mutation burden, was strongly correlated with total mutations, as expected. Similar results (not shown) were found in the Australian cohort. Use of total mutations in the prognostic model made no important difference to our results or conclusions.

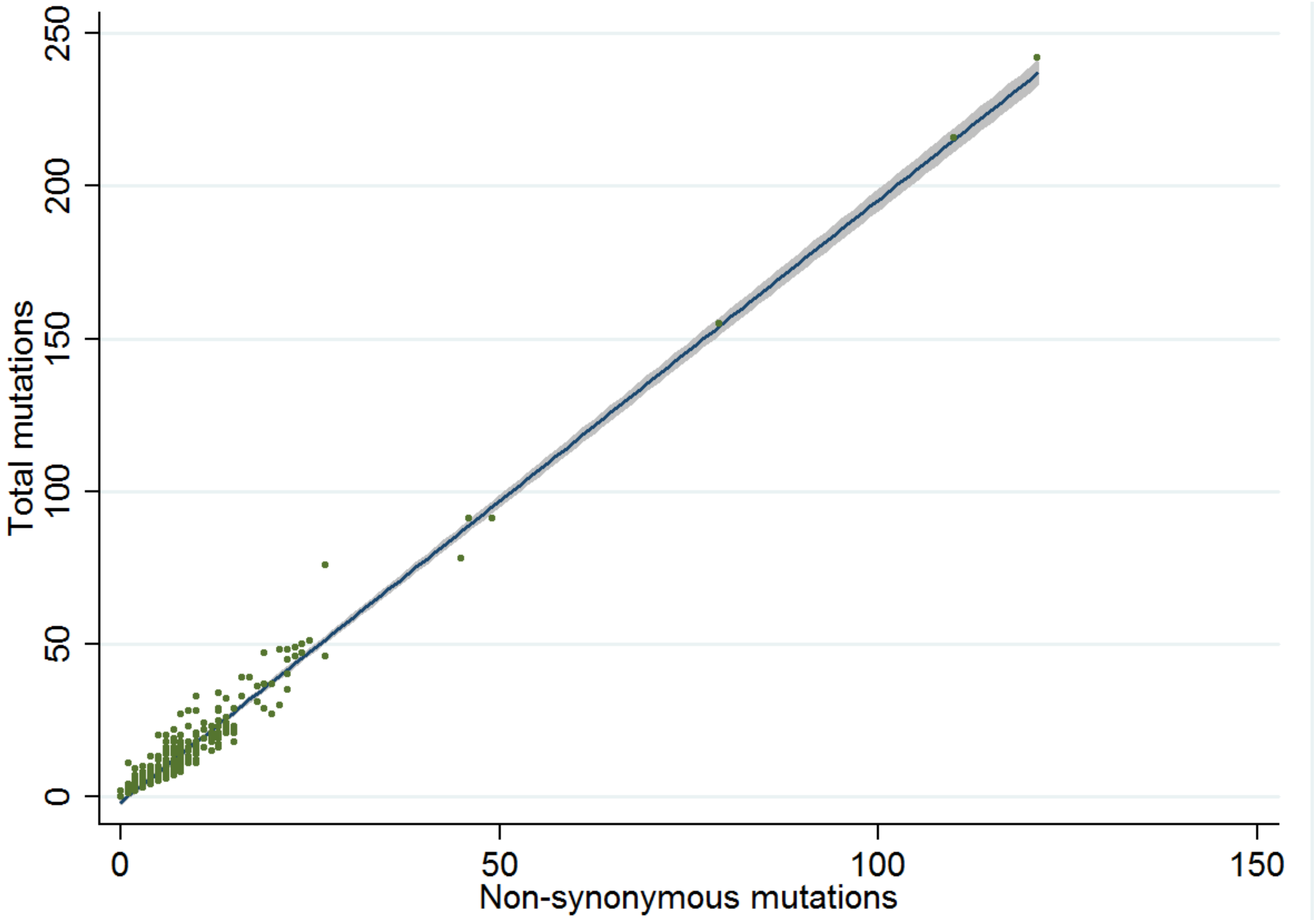

Supplementary References

1. Kerr RS, Love S, Segelov E, et al. Adjuvant capecitabine plus bevacizumab versus capecitabine alone in patients with colorectal cancer (QUASAR 2): an open-label, randomised phase 3 trial. The Lancet Oncology 2016; 17(11): 1543-57.
2. Boland CR, Goel A. Microsatellite instability in colorectal cancer. Gastroenterology 2010; 138(6): 2073-87.e3.
3. Domingo E, Freeman-Mills L, Rayner E, et al. Somatic POLE proofreading domain mutation, immune response, and prognosis in colorectal cancer: a retrospective, pooled biomarker study. Lancet Gastroenterol Hepatol 2016; 1(3): 207-16.
4. Palles C, Cazier J-B, Howarth KM, et al. Germline mutations affecting the proofreading domains of POLE and POLD1 predispose to colorectal adenomas and carcinomas. Nature genetics 2013; 45(2): 136-44.
5. Kerr RS, Love S, Segelov E, et al. Adjuvant capecitabine plus bevacizumab versus capecitabine alone in patients with colorectal cancer (QUASAR 2): an open-label, randomised phase 3 trial. Lancet Oncol 2016.
6. Pradhan M, Abeler VM, Danielsen HE, Trope CG, Risberg BA. Image cytometry DNA ploidy correlates with histological subtypes in endometrial carcinomas. Mod Pathol 2006; 19(9): 1227-35.
7. Merriman B, Ion Torrent R, Team D, Rothberg JM. Progress in ion torrent semiconductor chip based sequencing. Electrophoresis 2012; 33(23): 3397-417.
8. Hofreiter M, Jaenicke V, Serre D, von Haeseler A, Paabo S. DNA sequences from multiple amplifications reveal artifacts induced by cytosine deamination in ancient DNA. Nucleic Acids Res 2001; 29(23): 4793-9.
9. Cancer Genome Atlas N. Comprehensive molecular characterization of human colon and rectal cancer. Nature 2012; 487(7407): 330-7.
10. Alexandrov LB, Nik-Zainal S, Wedge DC, et al. Signatures of mutational processes in human cancer. Nature 2013; 500(7463): 415-21.
11. Palles C, Cazier JB, Howarth KM, et al. Germline mutations affecting the proofreading domains of POLE and POLD1 predispose to colorectal adenomas and carcinomas. Nat Genet 2013; 45(2): 136-44.
12. Chang MT, Asthana S, Gao SP, et al. Identifying recurrent mutations in cancer reveals widespread lineage diversity and mutational specificity. Nat Biotechnol 2016; 34(2): 155-63.
13. Bouaoun L, Sonkin D, Ardin M, et al. TP53 Variations in Human Cancers: New Lessons from the IARC TP53 Database and Genomics Data. Hum Mutat 2016; 37(9): 865-76.
14. Domingo E, Ramamoorthy R, Oukrif D, et al. Use of multivariate analysis to suggest a new molecular classification of colorectal cancer. The Journal of pathology 2013; 229(3): 441-8.
15. Tie J, Lipton L, Desai J, et al. KRAS mutation is associated with lung metastasis in patients with curatively resected colorectal cancer. Clinical cancer research : an official journal of the American Association for Cancer Research 2011; 17(5): 1122-30.
16. Rui Y, Wang C, Zhou Z, Zhong X, Yu Y. K-Ras mutation and prognosis of colorectal cancer: a meta-analysis. Hepatogastroenterology 2015; 62(137): 19-24.
17. Sinicrope FA, Shi Q, Smyrk TC, et al. Molecular markers identify subtypes of stage III colon cancer associated with patient outcomes. Gastroenterology 2015; 148(1): 88-99.
18. Phipps AI, Limburg PJ, Baron JA, et al. Association between molecular subtypes of colorectal cancer and patient survival. Gastroenterology 2015; 148(1): 77-87 e2.
19. Taieb J, Zaanan A, Le Malicot K, et al. Prognostic Effect of BRAF and KRAS Mutations in Patients With Stage III Colon Cancer Treated With Leucovorin, Fluorouracil, and Oxaliplatin With or Without Cetuximab: A Post Hoc Analysis of the PETACC-8 Trial. JAMA Oncol 2016: 1-11.
20. Andre T, de Gramont A, Vernerey D, et al. Adjuvant Fluorouracil, Leucovorin, and Oxaliplatin in Stage II to III Colon Cancer: Updated 10-Year Survival and Outcomes According to BRAF Mutation and Mismatch Repair Status of the MOSAIC Study. J Clin Oncol 2015; 33(35): 4176-87.
21. Mouradov D, Domingo E, Gibbs P, et al. Survival in stage II/III colorectal cancer is independently predicted by chromosomal and microsatellite instability, but not by specific driver mutations. The American journal of gastroenterology 2013; 108(11): 1785-93.
22. Kadowaki S, Kakuta M, Takahashi S, et al. Prognostic value of KRAS and BRAF mutations in curatively resected colorectal cancer. World J Gastroenterol 2015; 21(4): 1275-83.
23. Seppala TT, Bohm JP, Friman M, et al. Combination of microsatellite instability and BRAF mutation status for subtyping colorectal cancer. Br J Cancer 2015; 112(12): 1966-75.
24. Jorissen RN, Christie M, Mouradov D, et al. Wild-type APC predicts poor prognosis in microsatellite-stable proximal colon cancer. Br J Cancer 2015; 113(6): 979-88.

25. Merok MA, Ahlquist T, Royrvik EC, et al. Microsatellite instability has a positive prognostic impact on stage II colorectal cancer after complete resection: results from a large, consecutive Norwegian series. Ann Oncol 2013; 24(5): 1274-82.
26. Dienstmann R, Mason MJ, Sinicrope FA, et al. Prediction of overall survival in stage II and III colon cancer beyond TNM system: a retrospective, pooled biomarker study. Ann Oncol 2017; 28(5): 1023-31.
27. Schell MJ, Yang M, Teer JK, et al. A multigene mutation classification of 468 colorectal cancers reveals a prognostic role for APC. Nat Commun 2016; 7: 11743.
28. Deng Y, Wang L, Tan S, et al. KRAS as a predictor of poor prognosis and benefit from postoperative FOLFOX chemotherapy in patients with stage II and III colorectal cancer. Mol Oncol 2015; 9(7): 1341-7.
29. Manceau G, Marisa L, Boige V, et al. PIK3CA mutations predict recurrence in localized microsatellite stable colon cancer. Cancer Med 2015; 4(3): 371-82.
